# Compact optical convolution processing unit based on multimode interference

**DOI:** 10.1038/s41467-023-38786-x

**Published:** 2023-05-24

**Authors:** Xiangyan Meng, Guojie Zhang, Nuannuan Shi, Guangyi Li, José Azaña, José Capmany, Jianping Yao, Yichen Shen, Wei Li, Ninghua Zhu, Ming Li

**Affiliations:** 1 State Key Laboratory on Integrated Optoelectronics, Institute of Semiconductors, Chinese Academy of Sciences, 100083 Beijing, China; 2 Center of Materials Science and Optoelectronics Engineering, University of Chinese Academy of Sciences, 100190 Beijing, China; 3 School of Electronic, Electrical and Communication Engineering, University of Chinese Academy of Sciences, 100049 Beijing, China; 4 Institut National de la Recherche Scientifique—Énergie Matériaux et Télécommunications (INRS-EMT), H5A 1K6 Montréal, QC Canada; 5 ITEAM Research Institute, Universitat Politècnica de València, 46022 Valencia, Spain; 6 Guangdong Provincial Key Laboratory of Optical Fiber Sensing and Communications, Institute of Photonics Technology, Jinan University, 511443 Guangzhou, China; 7 Microwave Photonic Research Laboratory, School of Electrical Engineering and Computer Science, University of Ottawa, K1N 6N5 25 Templeton Street, Ottawa, ON Canada; 8 Lightelligence Group, 311121 Hangzhou, China

**Keywords:** Microwave photonics, Integrated optics

## Abstract

Convolutional neural networks are an important category of deep learning, currently facing the limitations of electrical frequency and memory access time in massive data processing. Optical computing has been demonstrated to enable significant improvements in terms of processing speeds and energy efficiency. However, most present optical computing schemes are hardly scalable since the number of optical elements typically increases quadratically with the computational matrix size. Here, a compact on-chip optical convolutional processing unit is fabricated on a low-loss silicon nitride platform to demonstrate its capability for large-scale integration. Three 2 × 2 correlated real-valued kernels are made of two multimode interference cells and four phase shifters to perform parallel convolution operations. Although the convolution kernels are interrelated, ten-class classification of handwritten digits from the MNIST database is experimentally demonstrated. The linear scalability of the proposed design with respect to computational size translates into a solid potential for large-scale integration.

## Abstract (web summary)

In most optical computing schemes, the size of the chip increases quadratically with the problem size. Here, the authors demonstrate an architecture for optical convolutional neural networks which, while losing the independent reconfigurability of the kernels, allows for linear scaling of the circuit size.

## Introduction

Inspired by the working mechanisms in biological visual nervous systems, convolutional neural networks (CNNs) have become a powerful category of artificial neural networks^1^. CNNs are commonly used in image recognition to greatly reduce the network complexity and conduct high-precision predictions, with wide applications in object classification, computer vision, real-time translation, and other areas^2–5^. As an increasing number of complex scenarios continue to emerge, including auto-driving and artificial intelligence services on the cloud^6,7^, it is strongly desired to increase the processing speed of the underlying neuromorphic hardware while reducing its computing energy consumption. However, in present schemes, mainly based upon the von Neumann computing paradigm, there is an inherent trade-off between the data exchange speed and the energy consumption; this is mainly because in these schemes, the memory and process unit are separated^8–11^.

Optical neural networks (ONNs) are regarded as promising candidates for the next generation of neuromorphic hardware processors. Photonics devices have low interconnect loss and can overcome the bandwidth bottleneck of their electrical counterparts to achieve ultrahigh computing bandwidth up to 10 THz^12–17^. Additionally, the light transmission in the ONN simultaneously implements data processing, which effectively avoids data tidal transmission in the von Neumann computing paradigm. In recent years, ONNs have attracted much interest in the realization of high-speed, large-scale and high-parallel optical neuromorphic hardware, with demonstrations including the use of light diffraction^18–24^, light interference^25–30^, light scattering^31,32^ and time-wavelength multiplexing^16,33–39^. The reported ONNs have been comparable to the state-of-the-art digital processors in terms of efficiency but have revealed a huge leap in computing density^40,41^. From the calculation results, ONN has the potential to improve at least two orders of magnitude in terms of energy consumption and computing density^42^. However, most of the reported works point to a quadratic increase in the component count, chip size and power consumption as the computational matrix size is scaled up^43^, which largely limits the integration potential of the resulting optical computing scheme while significantly increasing the complexity of the manipulation. The linearly scalable compact integrated diffractive optical network (IDNN) demonstrated in ref. ^24^. still requires 2*N* units to implement the input dimension of *N*.

In this paper, we propose a compact on-chip incoherent optical convolution processing unit (OCPU) integrated on a low-loss silicon nitride (SiN) platform to extract various feature maps in a fully parallel fashion. Leveraging on the combination of wavelength division multiplexing (WDM) technology and multimode interference coupling, the OCPU, includes two 4 × 4 multimode interference (MMI) cells and four phase shifters (PSs) as the minimum element count, can simultaneously support three 2 × 2 correlated real-valued kernels. Hence, three groups of convolution computing operations are performed in the OCPU in a parallel manner. The proposed unit is also dynamically reconfigurable only by tuning four PSs. Although the kernels are interrelated, the OCPU can work as a specific convolutional layer. The front-end SiN-based OCPU and an electrical fully connected layer jointly form a CNN, which is utilized to perform a ten-class classification operation from the Modified National Institute of Standards and Technology (MNIST)^44^ handwritten digits with an accuracy of 92.17%. Moreover, the components in the proposed OCPU grow linearly (N units for input dimension of N) with the size of the calculated matrix, providing solid potential for on-chip realization of OCPUs with increased computation capabilities, higher processing speed and lower power consumption toward the next generation of artificial intelligence platforms.

## Results

### Principle

**Fig. 1:**
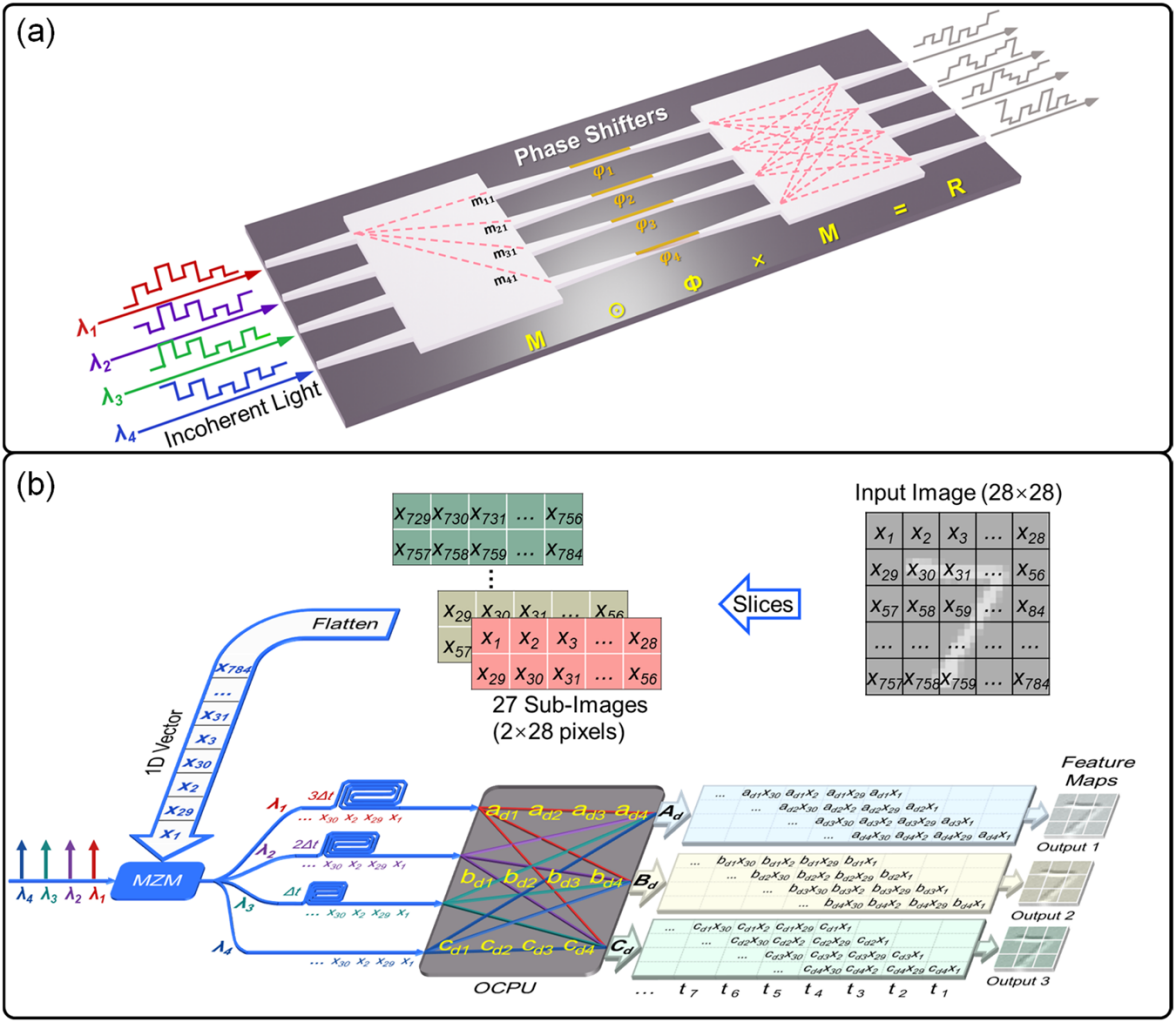
Convolution operation based on a compact optical convolutional processing unit (OCPU). **a** Structure diagram of OCPU. **b** The OCPU simultaneously performs three different groups of convolutional operations using incoherent light. The unit includes three functional parts: (1) input image slices to 27 sub-images; (2) flatten 27 sub-images into one-dimensional (1D) vectors; and (3) implement the convolutional operation with the OCPU.

The structure diagram of the designed OCPU is shown in Fig. 1a, which contains two 4 × 4 MMI cells and four PSs. The input data are encoded into four incoherent light waves and then sent into the OCPU to perform multiply accumulated (MAC) operations. The OCPU, as parallel multiple kernels, can simultaneously implement several groups of convolution operations. Each output port is regarded as an independent kernel, and the number of elements for each kernel is equal to that of the input ports, which indicates that the computing capability increases with the number of input ports. In addition, the kernel is dynamically reconfigurable by changing the current of the PSs via the thermo-optic effect.

As shown in Fig. 1a, the input vector $I$ is simultaneously modulated on the amplitude of four incoherent light waves with the same initial amplitudes via electro-optical modulation. The complex-valued transfer matrices $M$ and $\varPhi$ for an MMI cell and PS array, respectively, are written as:

1 $$\begin{array}{c}M=\left[\begin{array}{cc}\begin{array}{cc}{m}_{11} & {m}_{12}\\ {m}_{21} & {m}_{22}\end{array} & \begin{array}{cc}{m}_{13} & {m}_{14}\\ {m}_{23} & {m}_{24}\end{array}\\ \begin{array}{cc}{m}_{31} & {m}_{32}\\ {m}_{41} & {m}_{42}\end{array} & \begin{array}{cc}{m}_{33} & {m}_{34}\\ {m}_{43} & {m}_{44}\end{array}\end{array}\right]\end{array},\varPhi=\left[\begin{array}{cc}\begin{array}{cc}{e}^{j{\varphi }_{1}} & {e}^{j{\varphi }_{1}}\\ {e}^{j{\varphi }_{2}} & {e}^{j{\varphi }_{2}}\end{array} & \begin{array}{cc}{e}^{j{\varphi }_{1}} & {e}^{j{\varphi }_{1}}\\ {e}^{j{\varphi }_{2}} & {e}^{j{\varphi }_{2}}\end{array}\\ \begin{array}{cc}{e}^{j{\varphi }_{3}} & {e}^{j{\varphi }_{3}}\\ {e}^{j{\varphi }_{4}} & {e}^{j{\varphi }_{4}}\end{array} & \begin{array}{cc}{e}^{j{\varphi }_{3}} & {e}^{j{\varphi }_{3}}\\ {e}^{j{\varphi }_{4}} & {e}^{j{\varphi }_{4}}\end{array}\end{array}\right],$$

where the element ${m}_{{uv}}\left(u=1 \sim 4,v=1 \sim 4\right)$ in $M$ means the response of the MMI linking the output port $u$ and the input port $v$, and each row of $\varPhi$ is the additional phase of a PS. After transmission in the OCPU and square-law detection at the photodetectors (PDs), the full transfer matrix of OCPU can be expressed as:

2 $$\begin{array}{c}R=\left(M\times (\varPhi \odot M)\right)\odot \left(M\times (\varPhi \odot M)\right)=\left[\begin{array}{cc}\begin{array}{cc}{{{{{\rm{|}}}}}}{r}_{11}{{{{{{\rm{|}}}}}}}^{2} & {{{{{\rm{|}}}}}}{r}_{12}{{{{{{\rm{|}}}}}}}^{2}\\ {{{{{\rm{|}}}}}}{r}_{21}{{{{{{\rm{|}}}}}}}^{2} & {{{{{\rm{|}}}}}}{r}_{22}{{{{{{\rm{|}}}}}}}^{2}\end{array} & \begin{array}{cc}{{{{{\rm{|}}}}}}{r}_{13}{{{{{{\rm{|}}}}}}}^{2} & {{{{{\rm{|}}}}}}{r}_{14}{{{{{{\rm{|}}}}}}}^{2}\\ {{{{{\rm{|}}}}}}{r}_{23}{{{{{{\rm{|}}}}}}}^{2} & {{{{{\rm{|}}}}}}{r}_{24}{{{{{{\rm{|}}}}}}}^{2}\end{array}\\ \begin{array}{cc}{{{{{\rm{|}}}}}}{r}_{31}{{{{{{\rm{|}}}}}}}^{2} & {{{{{\rm{|}}}}}}{r}_{32}{{{{{{\rm{|}}}}}}}^{2}\\ {{{{{\rm{|}}}}}}{r}_{41}{{{{{{\rm{|}}}}}}}^{2} & {{{{{\rm{|}}}}}}{r}_{42}{{{{{{\rm{|}}}}}}}^{2}\end{array} & \begin{array}{cc}{{{{{\rm{|}}}}}}{r}_{33}{{{{{{\rm{|}}}}}}}^{2} & {{{{{\rm{|}}}}}}{r}_{34}{{{{{{\rm{|}}}}}}}^{2}\\ {{{{{\rm{|}}}}}}{r}_{43}{{{{{{\rm{|}}}}}}}^{2} & {{{{{\rm{|}}}}}}{r}_{44}{{{{{{\rm{|}}}}}}}^{2}\end{array}\end{array}\right],\end{array}$$

where the symbol $\odot$ is the Hadamard product^45^ (e.g., multiplication of the elements in the corresponding positions between matrix $M$ and matrix $\varPhi$) and the symbol $\times$ represents the multiplication of two matrices.

When a 4 × 1 vector $I$ is input to the OCPU, vector-matrix multiplication (VMM) is conducted in the OCPU, and the operation result is inferred as $O=R\times I$, where each output of the OCPU is the weighted summation of input vector $I$, which can be regarded as a convolutional result. Therefore, each row of $R$ can be used as a convolution kernel without negative values. Negative values are also achieved by setting any one vector as a ground line and subtracting it from the remaining three vectors. Taking the last vector as a ground line, for example, three kernels ${A}_{d} \sim {C}_{d}$ with negative values are rewritten as:

3 $$\begin{array}{c}{A}_{d}=\left[\begin{array}{cc}{{{{{\rm{|}}}}}}{r}_{11}{{{{{{\rm{|}}}}}}}^{2}-{{{{{\rm{|}}}}}}{r}_{41}{{{{{{\rm{|}}}}}}}^{2} & {{{{{\rm{|}}}}}}{r}_{13}{{{{{{\rm{|}}}}}}}^{2}-{{{{{\rm{|}}}}}}{r}_{43}{{{{{{\rm{|}}}}}}}^{2}\\ {{{{{\rm{|}}}}}}{r}_{12}{{{{{{\rm{|}}}}}}}^{2}-{{{{{\rm{|}}}}}}{r}_{42}{{{{{{\rm{|}}}}}}}^{2} & {{{{{\rm{|}}}}}}{r}_{14}{{{{{{\rm{|}}}}}}}^{2}-{{{{{\rm{|}}}}}}{r}_{44}{{{{{{\rm{|}}}}}}}^{2}\end{array}\right]=\left[\begin{array}{cc}{a}_{d1} & {a}_{d3}\\ {a}_{d2} & {a}_{d4}\end{array}\right],\\ {B}_{d}=\left[\begin{array}{cc}{{{{{\rm{|}}}}}}{r}_{21}{{{{{{\rm{|}}}}}}}^{2}-{{{{{\rm{|}}}}}}{r}_{41}{{{{{{\rm{|}}}}}}}^{2} & {{{{{\rm{|}}}}}}{r}_{23}{{{{{{\rm{|}}}}}}}^{2}-{{{{{\rm{|}}}}}}{r}_{43}{{{{{{\rm{|}}}}}}}^{2}\\ {{{{{\rm{|}}}}}}{r}_{22}{{{{{{\rm{|}}}}}}}^{2}-{{{{{\rm{|}}}}}}{r}_{42}{{{{{{\rm{|}}}}}}}^{2} & {{{{{\rm{|}}}}}}{r}_{24}{{{{{{\rm{|}}}}}}}^{2}-{{{{{\rm{|}}}}}}{r}_{44}{{{{{{\rm{|}}}}}}}^{2}\end{array}\right]=\left[\begin{array}{cc}{b}_{d1} & {b}_{d3}\\ {b}_{d2} & {b}_{d4}\end{array}\right],\\ {C}_{d}=\left[\begin{array}{cc}{{{{{\rm{|}}}}}}{r}_{31}{{{{{{\rm{|}}}}}}}^{2}-{{{{{\rm{|}}}}}}{r}_{41}{{{{{{\rm{|}}}}}}}^{2} & {{{{{\rm{|}}}}}}{r}_{33}{{{{{{\rm{|}}}}}}}^{2}-{{{{{\rm{|}}}}}}{r}_{43}{{{{{{\rm{|}}}}}}}^{2}\\ {{{{{\rm{|}}}}}}{r}_{32}{{{{{{\rm{|}}}}}}}^{2}-{{{{{\rm{|}}}}}}{r}_{42}{{{{{{\rm{|}}}}}}}^{2} & {{{{{\rm{|}}}}}}{r}_{34}{{{{{{\rm{|}}}}}}}^{2}-{{{{{\rm{|}}}}}}{r}_{44}{{{{{{\rm{|}}}}}}}^{2}\end{array}\right]=\left[\begin{array}{cc}{c}_{d1} & {c}_{d3}\\ {c}_{d2} & {c}_{d4}\end{array}\right].\end{array}$$

From Eqs. (1) and (3), the dynamically reconfigurable kernel matrix is implemented by tuning the PSs using the thermo-optic effect. This is based on the change that is induced on the refractive index of the waveguides with the driving current employed in the microheaters of the PSs, allowing light waves to acquire a desired extra phase. In Eq. (2), ${r}_{{uv}}$ changes with the phase of the optical waveform; therefore, ${A}_{d}$, ${B}_{d}$, and ${C}_{d}$ are subsequently changed with the phase to reconstruct three new kernels (more details can be seen in Supplementary Note 1).

The convolution process for feature map extraction is shown in Fig. 1b, which includes a serial data one-dimensional (1D) flattening operation, the optical kernel core representation and the convolution operation with the OCPU. First, the procedure of how to compress a two-dimensional (2D) image matrix into a 1D vector is shown in Fig. 1b. Taking a “7” digital image with 28 × 28 pixels as an example, the 28 × 28 matrix is totally divided into 27 sub-matrix slices along the longitudinal axis, with 2 × 28 elements for each sub-image. Then, the 27 sub-images are flattened by column into sub-vectors and form a 1 × 1512 vector $$X=[\begin{array}{ccccccc}{x}_{1} & {x}_{29} & {x}_{2} & {x}_{30} & \cdots & {x}_{756} & {x}_{784}\end{array}]$$ by means of connecting the sub-vectors head-to-tail.

The sequential data $X$ simultaneously modulate the amplitude of incoherent light waves with wavelengths of ${\lambda }_{1} \sim {\lambda }_{4}$ via the Mach–Zehnder modulator (MZM) and generate four replicas of encoded data $X$. Then, the optical waveforms are routed into four parallel channels with one wavelength in each and undergo a time delay of $\varDelta \tau$ between adjacent channels, equal to the reciprocal of the baud rate of the modulation signal ${f}_{b}$ (i.e., $\varDelta \tau=\frac{1}{{f}_{b}}$). Four temporal waveforms are reallocated and recombined at the output port of the OCPU. The orthogonality between each channel is guaranteed by the incoherent beam, such that different input waveforms propagate individually in the OCPU. Subsequently, the PD implements square-law detection and sums the power of the four incoherent wavelengths (the relationship between the bandwidth of the PD and the wavelength interval of incoherent wavelengths is further discussed in Supplementary Note 6). The computing result at each time slot of each output port is the convolution between the adjacent four elements in vector $X$ and the 2 × 2 kernel matrix ${A}_{d}$, ${B}_{d}$, or ${C}_{d}$.

Some insignificant values are contained in the output of OCPU, which need to be eliminated to achieve feature extraction following the principle of convolution operation. The rule to retain the effective elements in the convolution results is that the even-numbered values except the first one are significant for each sub-vector. Hence, for the first sub-vector, the 27 effective values in the first row of the feature matrix are $$\left[\begin{array}{cc}\begin{array}{cc}{y}_{4} & {y}_{6}\end{array} & \begin{array}{cc}\cdots & {y}_{56}\end{array}\end{array}\right]$$. Finally, 27 rows of effective values are rearranged in a column format to form the 27 × 27 feature matrix with a kernel sliding window of 1 (more details can be seen in Supplementary Note 2).

The OCPU is able to simultaneously perform a multi-kernel parallel convolution operation. From Fig. 1b, each output port works as a 1 × 4 weight vector or a 2 × 2 kernel, and 4 MAC operations are performed at each time slot. Therefore, the computing speed is equal to $4{f}_{b}$ MAC operations per second for each output port. The total computing speed of the OCPU with three parallel kernels is thereby $3\times 4{f}_{b}=12{f}_{b}$ MAC operations per second. In general, for an OCPU with $n$ input/output ports, the total computing speed reaches $n\left(n-1\right){f}_{b}$ MAC operations per second. In summary, the computing speed of MAC operations for one port is linearly proportional to the number of elements in a kernel, and the overall computing capability of OCPU increases quadratically with the parallel scale. It is worth noting that there is a certain correlation of the formed $n-1$ kernels in the OCPU. The reconfiguration of one kernel inevitably results in linkage to other kernels (this is discussed in more detail in Supplementary Note 11).

### The OCPU chip

**Fig. 2:**
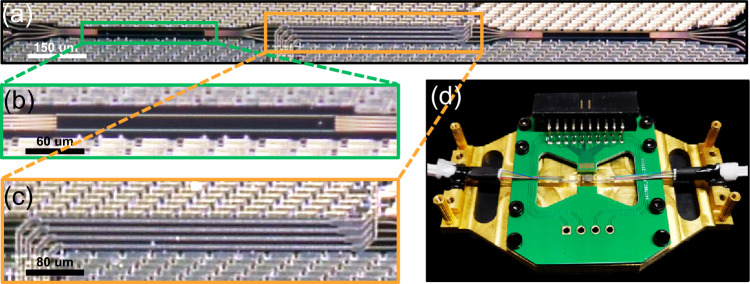
Image of the convolution processor. **a** Microscope image of the OCPU chip with two 4 × 4 MMI cells and four PSs. **b** Microscope image of the MMI. **c** Microscope image of the phase shift region. **d** The packaged chip.

The SiN-based OCPU, as the parallel convolution kernel, is fabricated at a CMOS compatible platform using the low-pressure chemical vapor deposition and Damascus process to realize the low-loss and high-confinement SiN waveguides^46^. The micrographs of the chip are shown in Fig. 2a–c, where Fig. 2a is the microscope image of the whole OCPU, Fig. 2b shows the microscope image of the 4 × 4 MMI cell, which features a footprint of 275 µm × 15 µm and an insertion loss of ~1 dB, and Fig. 2c shows the phase shift region based on the thermo-optic effect. The transition waveguides between the multimode regions and the straight waveguide are tapers with a linearly varying width from 2 to 1 µm to reduce the scattering loss from the sharp edges. The PSs between the two MMI cells are covered with aluminum microheaters 400 µm in length, 1.5 µm in width and 0.4 µm in height. Spot size converters at input/output facets are coupled with standard single mode fibers with an edge coupling loss of ~1.5 dB per port. Figure 2d shows the packaged OCPU.

### Experiment

**Fig. 3:**
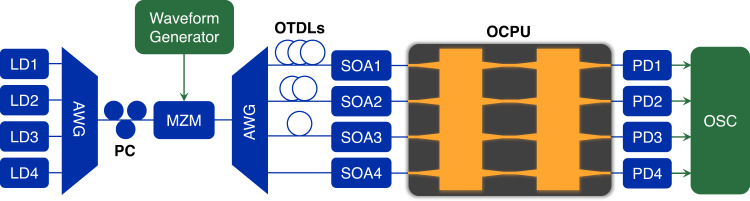
Experimental setup of convolution computing with the OCPU. LD laser diode, AWG arrayed waveguide grating, PC polarization controller, OTDL optical tunable delay line, SOA semiconductor optical amplifier, PD photodetector, OSC oscilloscope.

Here, we experimentally demonstrate the optical convolution operation to extract the feature maps of handwritten digits with the proposed layout shown in Fig. 3. Four wavelength-dependent light waves are generated from the laser array with wavelengths of 1549.32, 1550.12, 1550.92, and 1551.72 nm and then multiplexed in an arrayed waveguide grating (AWG) to simultaneously achieve electro-optical conversion in a Mach–Zehnder modulator (MZM). Here, the data rate from the waveform generator is set to 16.60 Gbaud/s (each data point is sampled 3 times with a sampling rate of 49.8 GSa/s), corresponding to a fixed delay of $1\div16.60G\approx 60.24$ ps. Afterward, the temporal waveforms underwent wavelength-division demultiplexing and time delay with three optical tunable delay lines (OTDLs) to reach a one-bit time delay between adjacent channels. Four semiconductor optical amplifiers (SOAs) are used to compensate for the loss along each channel. After summing the replicas from the output port of the OCPU, the powers of the incoherent beam are converted into electrical signals by PDs and recorded by an oscilloscope (OSC).

**Fig. 4:**
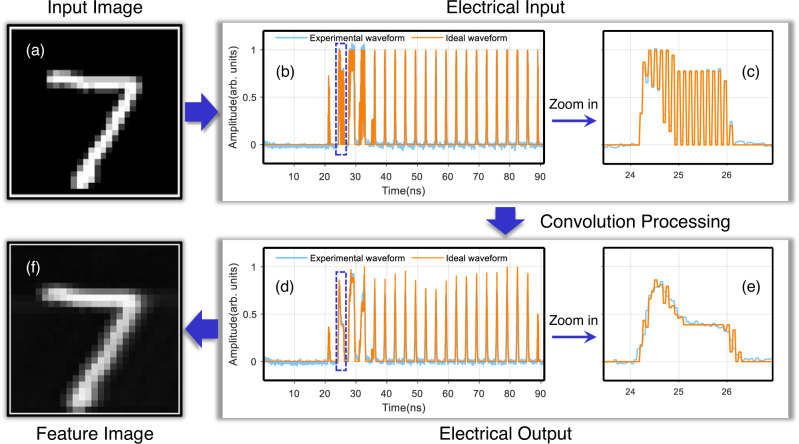
Experimental demonstration of digital “7”. **a** The input image of digit “7” from the MNIST database. **b** Sequential ideal (orange line) and experimental (blue line) electronic waveforms. **d** Ideal and experimental convolution output waveform with the kernel of $$\left[\begin{array}{cc}1 & 0\\ 0 & 1\end{array}\right]$$. **c**, **e** are magnifications of (**b**) and (**d**) at 23.43–26.95 ns, respectively. **f** The recovered feature image.

The computing performance of the OCPU is first verified by extracting the feature map of handwritten digits with 28 × 28 pixels and 8-bit resolution from the MNIST handwritten digits database. Figure 4 shows the convolution process of digit “7” with the kernel of $$\left[\begin{array}{cc}1 & 0\\ 0 & 1\end{array}\right]$$. The image is first flattened into a 1 × 1512 (i.e., $1512=(2\times 28)\times 27$) vector, where (2 × 28) represents the number of elements for each sub-matrix and 27 is the number of submatrices. Then, the 1 × 1512 vector is encoded into a serial electrical waveform from the waveform generator and fed into the MZM to modulate the intensity of the light wave at a data rate of 16.60 Gbaud/s. Therefore, the convolution time with a non-negative kernel is $1512\div16.60\,\approx \,91.08$ ns for one image, that is, $1\div91.08{{{{{{\rm{ns}}}}}}}\,\approx \,10.98$ million images per second (multiple acquisitions are needed to reduce noise when kernels contain negative values). Figure 4a is the input image of digit “7” from the MNIST database, and Fig. 4b shows the ideal waveform of the flattened digit “7” (orange line) and the experimental one (blue line) from the waveform generator. Figure 4d shows the ideal and experimental convolution results, and the feature image in Fig. 4f is recovered from significant values in Fig. 4d. Figure 4c, e shows magnified images of Fig. 4b, d at 23.43–26.95 ns, respectively.

**Fig. 5:**
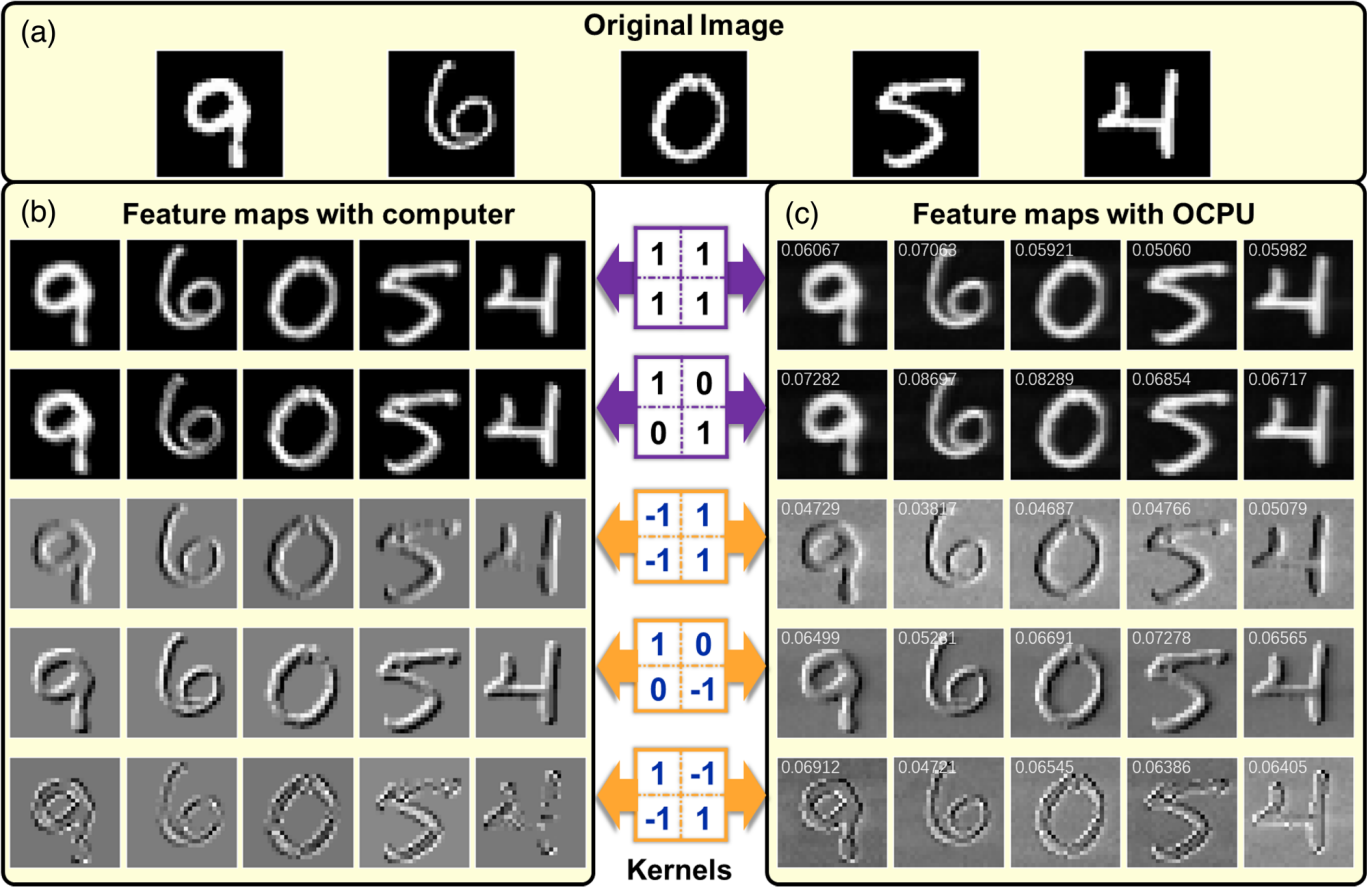
Feature images with reconfigurable kernels. **a** The input handwritten digit images of “9”, “6”, “0”, “5”, and “4”. **b** Feature maps with the digital computer. **c** Feature maps with the OCPU chip. The numbers marked on each picture are the root mean square error (RMSE) between feature maps obtained with the computer and the OCPU.

The kernel of the OCPU is dynamically reconfigured by tuning the driving current of the PSs. In the experiment, kernels without negative values are acquired in a single output port for a single acquisition, and kernels involving both non-negative and negative values are achieved by subtracting the reference port from other ports and averaging 13 acquisitions to reduce noise. Figure 5 shows the original images (Fig. 5a) of five randomly selected MNIST digit images (“9”, “6”, “0”, “5” and “4”) as well as feature maps obtained with the digital computer (Fig. 5b) and the OCPU (Fig. 5c). Comparing the simulation results in the computer with the experimental results of the OCPU, the feature images extracted with the proposed OCPU fit well with the simulated results, with an average root mean square error (RMSE) of only 0.0617 among the 25 feature images shown in Fig. 5. The bit precision of MAC operations with the OCPU is also calibrated, and the standard deviation is −0.0298, resulting in a bit precision of 5-bit (more details about RMSE and bit precision can be seen in Supplementary Note 4).

Here, the sliding speed of the convolution window is equal to the encoded band rate of 16.60 Gbaud/s. Each output symbol is the result of 4 (the length of each kernel) MAC, and the computing speed is $4\times 16.60G=66.40$ giga-MAC operations per second for each kernel. For 3 real-value correlated kernels parallel accelerated computation in the OCPU, the total computing speed is up to $66.40\times 3=199.20$ giga-MAC operations per second. In the case of using four non-negative-value correlated kernels, the computing speed amounts to $66.40\times 4=265.60$ giga-MAC operations per second. In this work, the 28 × 28 pixel image is convolved with a 2 × 2 kernel to achieve a 27 × 27 pixel feature map, so the effective computing speed is $729\div1512\times 265.60=128.06$ giga-MAC operations per second, where the convolution results of each image are comprised of 1512 sample points and 729 significant values.

**Fig. 6:**
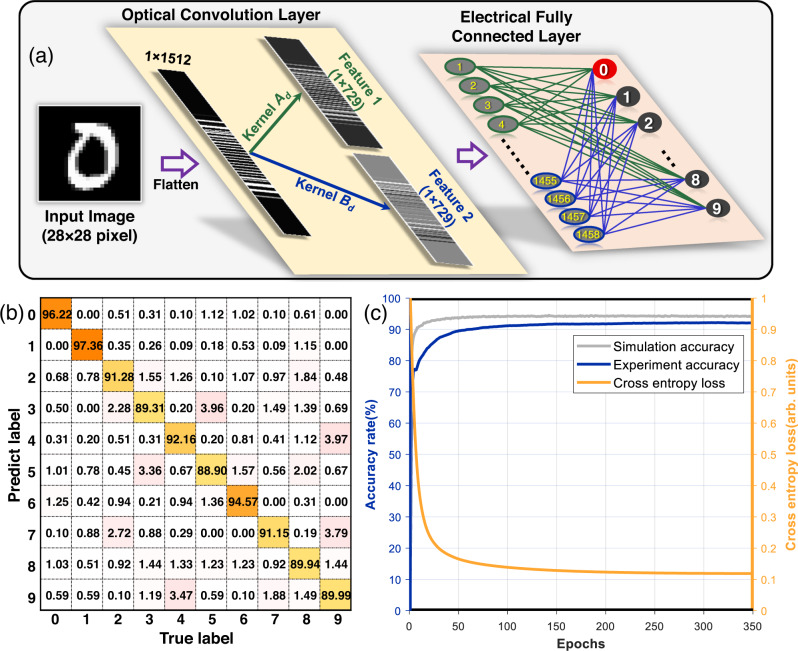
MNIST handwritten digital image classification demonstration. **a** The network structure of the CNN, which contains an optical convolution layer and an electrically fully connected layer. **b** The confusion matrix of recognizing 10,000 digits in the MNIST test database, where the abscissa indicates the true labels and the ordinate indicates the recognition results. **c** The variation in simulation accuracy, experimental accuracy, and experimental cross entropy loss during 350 epochs of training.

In Fig. 6a, we use the OCPU incorporating an electronic fully connected layer and a ReLU nonlinear activation function^47^ in a digital computer to form a CNN for the ten-class classification of “0 ~ 9” handwritten digit images. Two kernels are utilized in the optical convolution layer, generating two 1 × 729 feature maps. After being activated using the ReLU nonlinear function, two 1 × 729 feature maps are reshaped into a 1 × 1458 vector and then fed to the fully connected layer to implement the recognition task. Here, for the ten-class classification, the weight matrix of the fully connected layer with a size of 1458 × 10 is trained offline to converge on the minimum cross entropy loss using the backpropagation algorithm^48^ (stochastic gradient descent algorithm^49,50^). Therefore, ten output neurons are the result of matrix multiplication between the 1 × 1458 vector and weight matrix 1458 × 10, where the largest value of the 1 × 10 output represents the predicted category.

We experimentally demonstrate ten-class classification of 70,000 images from the MNIST dataset with 60,000 for training and 10,000 for testing. The confusion matrix for 10,000 test images (Fig. 6b) and the variation in classification accuracy (Fig. 6c) show an accuracy of 92.17% for the experiment versus 94.51% for the theory after 350 epochs. The deviation from the theoretical accuracy of 2.34% is mainly caused by the limited bit precision (the relationship between the bit precision and the recognition accuracy can be seen in Supplementary Note 9), which is caused by numerous factors, including the electrical and optical noise and instability of some optical devices (polarization state jitter, temperature drift). In addition, to work in the linear amplification region, low optical power is input to the low gain and high noise figure SOA, which makes it difficult to avoid introducing noise and leads to a low signal noise ratio at the PD. Moreover, digital domain processes such as analog-to-digital conversion and subtraction further raise the noise and degrade the signal-to-noise ratio. The average operation used in the experiment reduces the noise to a certain extent but at the cost of prolonging the calculation time. Balanced detection is an alternative scheme to dispel noise and improve bit precision without electrical average processing (an analysis of the further improvement in accuracy can be seen in Supplementary Note 10).

Table 1 presents a performance comparison of the representative computing framework, including the optical solutions (such as the Mach‒Zehnder interferometer (MZI)^25^, microring resonator (MRR)^33,51^, integrated diffractive optical network (IDNN)^24^, phase changed material (PCM)^16^ and others^35,52–54^) and analog electrical solution^55^. The programmable units in refs. ^16,24,25,33,51,53,54^. show quadratic relationship with the computational matrix size scaling, whereas the optical scheme has a linear relationship^24^ with a slope of 2. The programmable units in the OCPU grow linearly with the kernel size, and half of the components are purely needed to perform the equivalent computational scale in comparison to the linear relationship optical scheme^24^. Owing to the large reduction in the basic unit, the energy efficiency is calculated as 4.84 pJ/MAC, and the computational density is calculated as 12.74 TMACs/s/mm^2^ (more details can be seen in Supplementary Note 8). The OCPU offers a solution of high computational density at the slight cost of recognition accuracy. The strength of linear scalability will be greatly demonstrated with the figure of merit of computational density to a larger scale. Drawing the 4 × 4 chip design thought, the Si-based 9 × 9 chip size is estimated to be 0.0166 mm^2^, and the energy efficiency is expected to be 0.95 pJ/MAC. Consequently, the computed density is calculated to be 1.19 PMACs/s/mm^2^, which is a two-order-of-magnitude improvement over other optical solutions. (More designed details about the Si-based 9 × 9 OCPU can be seen in Supplementary Notes 7, 8 and 12).

**Table 1:** Performance comparison of our proposed OCPU framework

Type	Programmable units	Matrix dimension	Platform	Accuracy on MNIST test set	Network architecture	Efficiency (/MAC)	Precision of results	Compute density (MACs/s/mm^2^)	Scale
TOPS-CA^35^	/	9 × 10	/	88.00%	1Conv. (3 5 × 5 kernels) + 1FC	1.58 pJ	7-bit	System	/
Netcast^52^	/	/	/	98.80%	3FC	10.00 fJ^e^	8-bit	System	/
AOM-VMM^53^	3	1 × 3	/	98.90%	2Conv. (16 3 × 3 kernels) + 2FC	/	/	System	N^2^
MZI-VMM^25^	60	4 × 4	Si	76.70% (4 categories, vowel recognition)	2FC	30.00 fJ^a^	5-bit	0.56 T^a^	N^2^
MRR-VMM^33^	16	4 × 4	Si	/	/	0.18 pJ^b^	4-bit^f^	1.60 T^b^	N^2^
MRR-VMM^51^	4	1 × 4	Si	97.41%	3FC	0.56 pJ^b^	4-bit	2.89 T^b^	N^2^
PCM-VMM^16^	36	9 × 4	SiN	95.30%	1Conv. (4 2 × 2 kernels) + 1FC	5.00 pJ	7-bit^d^	0.60 T	N^2^
	64	8 × 8	Si	/	/	4.00 pJ	7-bit^d^	81.00 T	N^2^
PMMC-VMM^54^	4	1 × 4	SiN	91.00% (2 categories)	1Conv. (2 2 × 2 kernels) + 1FC	/	6-bit^f^	82.00 T^b^	N^2^
IDNN-VMM^24^	20	10 × 10^c^	Si	89.40%	2FC	/	/	/	2N
Flash (analog electronics, simulation)^55^	/	100 × 100	Si	/	/	7.00 fJ	5-bit	18.00 T	/
This work	4	4 × 4^c^	SiN	92.17%	1Conv. (2 2 × 2 kernels) + 1FC	4.84 pJ	5-bit	12.74 T	N
Expected from this work	9	9 × 9^c^	Si	96.35%	1Conv. (8 3 × 3 kernels) + 1FC	0.95 pJ	5-bit	1.19 P	N

^a^These data can be obtained based on existing state-of-the-art equipment.

^b^Data derived from a large-scale outlook of the proposed structure.

^c^The rows in the matrix are correlated to each other.

^d^For comparison under the same standard, the precision is recalculated following the standard deviation listed in the paper.

^e^Energy efficiency of the client.

^f^Precision of weight adjustment.

Although the OCPU-based architecture offers some advantages in computational density and so on, the correlation between kernels will limit the performance of the OCPU-based convolutional layer to some extent. Even so, the OCPU can still serve as a specific convolutional layer and significantly improve the recognition accuracy (more details can be seen in Supplementary Note 12). In scenarios such as edge computing, it may be sufficient to achieve reasonable performance given the strict restrictions on footprint or energy. In the future, exploring special application scenarios where this correlation does not affect performance will be an important research direction.

## Discussion

In summary, we have designed and demonstrated a SiN-based compact OCPU to extract various feature images. The demonstrated OCPU, includes two 4 × 4 multimode interference cells and four PSs and simultaneously performs a convolutional operation with three correlated, user-defined 2 × 2 real-valued kernels. Dynamic reconfiguration to extract the desired feature images is easily realized by tuning the PSs. The front-end SiN-based OCPU as well as an electrical fully connected layer form a CNN that enables efficient ten-class classification of MNIST handwritten digits. Owing to the phase regulatory mechanism, the proposed scheme offers numerous important advantages over previous designs, such as a compact size, easier manipulation and higher robustness. In addition, benefitting from the linear relationship between the number of elements and the dimension of the matrix, the proposed OCPU has solid potential for on-chip large-scale integration by simply increasing the number of ports as well as by utilizing a wavelength multiplexing strategy in each port toward the next generation of high-performance, ultrahigh-speed artificial intelligence platforms.

## Methods

### Configuration

Optical convolution computing with the proposed OCPU was implemented using commercially available optoelectronic components. The laser array is an IDPHOTONICS CoBrite-DX laser source with four tunable polarization-maintaining output ports to generate four wavelengths of 1549.32, 1550.12, 1550.92, and 1551.72 nm. Two AWGs are standard AWGs for communication from SHIJIA PHOTONS with a wavelength interval of 100 GHz (AAWG-F20-100) to couple four wavelengths into one beam and then wavelength-division demultiplexing into four beams after modulation in the MZM. The polarization controller (PC) is a Thorlabs FPC032 to adjust the polarization of the light beam. The MZM is an iXblue intensity modulator with a bandwidth of 40 GHz. The waveform generator is Tektronix AWG70001A with a maximum sample rate of 50 GSa/s to generate the input waveform. Three OTDLs are Advanced Fiber Resources VDL-1550-500 with a maximum delay of 500 ps to realize a 1-bit time delay between adjacent channels. The SOAs that are utilized to compensate for the loss of each channel are Thorlabs SOA10103S with a linear amplification area of ~22 dB. The PDs are Finisar XPDV2150R with a bandwidth of 50 GHz to convert optical waveforms into electrical waveforms. The temporal waveforms are sampled with a real-time oscilloscope (Tektronix DPO73304D).

## Supplementary information

Supplementary Information

## Data Availability

The data that support the findings of this study are available from the corresponding authors upon request. Source data are provided with this paper.

## Source data

Source Data
