# Supplementary material for: Compact optical convolution processing unit based on multimode interference
Source: Nat Commun. 2023 May 24;14:3000. doi: 10.1038/s41467-023-38786-x (PMC10209208; doi:10.1038/s41467-023-38786-x)
Supplement: Supplementary file 1 — Supplementary Information [file 41467_2023_38786_MOESM1_ESM.pdf]

Supplementary Information for

Compact optical convolution processing unit based on incoherent multimode interference

Compact optical convolution processing unit based on incoherent multimode interference

Authors:

Xiangyan Meng[1,2,3,#], Guojie Zhang[1,2,3,#], Nuannuan Shi[1,2,3*], Guangyi Li[1,2,3], José Azaña[4], José Capmany[5], Jianping Yao[6,7], Yichen Shen[8], Wei Li[1,2,3], Ninghua Zhu[1,2,3], Ming Li[1,2,3*]

Affiliations:

[1] State Key Laboratory on Integrated Optoelectronics, Institute of Semiconductors, Chinese Academy of Sciences, Beijing 100083, China

[2] Center of Materials Science and Optoelectronics Engineering, University of Chinese Academy of Sciences, Beijing 100190, China

[3] School of Electronic, Electrical and Communication Engineering, University of Chinese Academy of Sciences, Beijing 100049, China

[4] Institut National de la Recherche Scientifique – Énergie, Matériaux et Télécommunications (INRS-EMT), Montréal H5A 1K6, Canada

[5] ITEAM Research Institute, Universitat Politècnica de València, Valencia 46022, Spain

[6] Guangdong Provincial Key Laboratory of Optical Fiber Sensing and Communications, Institute of Photonics Technology, Jinan University, Guangzhou 511443, China

[7] Microwave Photonics Research Laboratory, School of Electrical Engineering and Computer Science, University of Ottawa, Ottawa, Ontario K1N 6N5, Canada

[8] Lightelligence Group, Hangzhou 311121, China

[#] Equal contributors.

E-mail:

*e-mail: nnshi@semi.ac.cn and ml@semi.ac.cn

Table of Contents

Supplementary Note 1: Kernel representation with OCPU 4
Supplementary Note 2: Significant convolution results extraction 7
Supplementary Note 3: Convolution kernel experimental implementation and temporal waveforms 8
Supplementary Note 4: Convolution error and bit precision 10
Supplementary Note 5: Convolution kernel reconstruction ability 11
Supplementary Note 6: Computing speed improvement 12
Supplementary Note 7: Simulation for 9×9 OCPU 13
Supplementary Note 8: Energy efficiency and compute density 14
Supplementary Note 9: Relationship between bit precision and recognition accuracy 16
Supplementary Note 10: Analysis of recognition accuracy 16
Supplementary Note 11: The limitations of convolution kernels 17
Supplementary Note 12: Handwritten digit recognition with 9×9 OCPU 19
Supplementary Note 13: Electrical overhead for the CNN 20
Supplementary References 33

Supplementary Note 1: Kernel representation with OCPU

When conducting the convolution operation with the proposed OCPU, the input vector is simultaneously modulated on the amplitude of four incoherent light waves with the same initial amplitudes via electro-optical modulation. The intensity of light waveforms after modulation is written as a real-valued vector I:

$$I = \begin{bmatrix} i_1 \\ i_2 \\ i_3 \\ i_4 \end{bmatrix}, \tag{1}$$

where the four elements are wavelength-dependent and enter the OCPU by four input ports. Complex-valued transfer matrices M and Φ for an MMI cell and phase shifter array are written as

$$M = \begin{bmatrix} m_{11} & m_{12} & m_{13} & m_{14} \\ m_{21} & m_{22} & m_{23} & m_{24} \\ m_{31} & m_{32} & m_{33} & m_{34} \\ m_{41} & m_{42} & m_{43} & m_{44} \end{bmatrix}, \Phi = \begin{bmatrix} e^{j\varphi_1} & e^{j\varphi_1} & e^{j\varphi_1} & e^{j\varphi_1} \\ e^{j\varphi_2} & e^{j\varphi_2} & e^{j\varphi_2} & e^{j\varphi_2} \\ e^{j\varphi_3} & e^{j\varphi_3} & e^{j\varphi_3} & e^{j\varphi_3} \\ e^{j\varphi_4} & e^{j\varphi_4} & e^{j\varphi_4} & e^{j\varphi_4} \end{bmatrix}, \tag{2}$$

where the element $m_{uv}\left(u = 1 \sim 4, v = 1 \sim 4\right)$ in M means the response of the MMI at output port u and input port v, and each row of Φ is the additional phase of a phase shifter. Thus, the full transfer matrix of OCPU is inferred as

$$R' = M \times \left(\Phi \odot M\right) = \begin{bmatrix} r_{11} & r_{12} & r_{13} & r_{14} \\ r_{21} & r_{22} & r_{23} & r_{24} \\ r_{31} & r_{32} & r_{33} & r_{34} \\ r_{41} & r_{42} & r_{43} & r_{44} \end{bmatrix}, \tag{3}$$

where $\odot$ is the Hadamard product[1] (e.g., multiplication of the elements in the corresponding positions between matrix M and matrix Φ) and $\times$ is the multiplication of two matrices. Therefore, the elements r_{uv} ($u = 1 \sim 4$, $v = 1 \sim 4$) are expressed as

$$r_{uv} = \sum\nolimits_{n=1}^{4} m_{un} m_{nv} e^{j\varphi_n}. \tag{4}$$

After square-law detection at the photodetectors (PDs), the transfer matrix of the OCPU is written as

$$R = R' \odot R' = \begin{bmatrix} |r_{11}|^2 & |r_{12}|^2 & |r_{13}|^2 & |r_{14}|^2 \\ |r_{21}|^2 & |r_{22}|^2 & |r_{23}|^2 & |r_{24}|^2 \\ |r_{31}|^2 & |r_{32}|^2 & |r_{33}|^2 & |r_{34}|^2 \\ |r_{41}|^2 & |r_{42}|^2 & |r_{43}|^2 & |r_{44}|^2 \end{bmatrix}. \tag{5}$$

The output from the OCPU is inferred as

$$O = R \times I = \begin{bmatrix} o_1 \\ o_2 \\ o_3 \\ o_4 \end{bmatrix}, \tag{6}$$

where each element represents one output from the OCPU, which can be expressed as follows:

$$o_u = \sum_{n=1}^{4} i_n |r_{un}|^2. \tag{7}$$

As shown in Eq. (7), each output o_u as a convolutional result is the weighted summation of input vector I. Therefore, each row of matrix R is set as a convolution kernel. As a result, four kernels without negative values are independently achieved as follows:

$$\begin{aligned} A &= \left[|r_{11}|^2 \quad |r_{12}|^2 \quad |r_{13}|^2 \quad |r_{14}|^2 \right], \\ B &= \left[|r_{21}|^2 \quad |r_{22}|^2 \quad |r_{23}|^2 \quad |r_{24}|^2 \right], \\ C &= \left[|r_{31}|^2 \quad |r_{32}|^2 \quad |r_{33}|^2 \quad |r_{34}|^2 \right], \\ D &= \left[|r_{41}|^2 \quad |r_{42}|^2 \quad |r_{43}|^2 \quad |r_{44}|^2 \right]. \end{aligned} \tag{8}$$

From Eq. (8), four 1×4 vectors $A \sim D$ are rearranged to form 2×2 convolution kernels $A' \sim D'$ as follows:

$$A' = \begin{bmatrix} |r_{11}|^2 & |r_{13}|^2 \\ |r_{12}|^2 & |r_{14}|^2 \end{bmatrix}, B' = \begin{bmatrix} |r_{21}|^2 & |r_{23}|^2 \\ |r_{22}|^2 & |r_{24}|^2 \end{bmatrix}, C' = \begin{bmatrix} |r_{31}|^2 & |r_{33}|^2 \\ |r_{32}|^2 & |r_{34}|^2 \end{bmatrix}, D' = \begin{bmatrix} |r_{41}|^2 & |r_{43}|^2 \\ |r_{42}|^2 & |r_{44}|^2 \end{bmatrix}. \quad (9)$$

From $A' \sim D'$ in Eq. (9), only positive values exist in the kernel matrix. Negative values are achieved by setting one matrix as a ground line and subtracting the ground line matrix from the other matrices. For example, we set the matrix D' as a ground line, and three brand-new kernels $A_d \sim C_d$ with negative parameters are obtained by subtracting the ground-line matrix D' from matrices $A' \sim C'$:

$$\begin{aligned} A_d &= \begin{bmatrix} |r_{11}|^2 - |r_{41}|^2 & |r_{13}|^2 - |r_{43}|^2 \\ |r_{12}|^2 - |r_{42}|^2 & |r_{14}|^2 - |r_{44}|^2 \end{bmatrix} = \begin{bmatrix} a_{d1} & a_{d3} \\ a_{d2} & a_{d4} \end{bmatrix}, \\ B_d &= \begin{bmatrix} |r_{21}|^2 - |r_{41}|^2 & |r_{23}|^2 - |r_{43}|^2 \\ |r_{22}|^2 - |r_{42}|^2 & |r_{24}|^2 - |r_{44}|^2 \end{bmatrix} = \begin{bmatrix} b_{d1} & b_{d3} \\ b_{d2} & b_{d4} \end{bmatrix}, \\ C_d &= \begin{bmatrix} |r_{31}|^2 - |r_{41}|^2 & |r_{33}|^2 - |r_{43}|^2 \\ |r_{32}|^2 - |r_{42}|^2 & |r_{34}|^2 - |r_{44}|^2 \end{bmatrix} = \begin{bmatrix} c_{d1} & c_{d3} \\ c_{d2} & c_{d4} \end{bmatrix}. \end{aligned} \quad (10)$$

From Eq. (4) and Eq. (10), since the matrix M is fixed with the fabricated MMI chip, the dynamically reconfigurable kernel matrix is implemented by tuning the phase shifters on the thermo-optic effect. The refractive index of waveguides changes with the driving current employed in the microheaters of the phase shifters, allowing light waves to attach an extra phase. In Eq. (4), r_{uv} change with the phase of the optical waveform; therefore, A_d, B_d and C_d are subsequently changed with the phase to reconstruct three new kernels.

Moreover, as observed, the matrix D' varies with the phase introduced by the phase shifters and is unable to serve as a global reference. Despite the absence of a global reference, incorporating the entire OCPU model into the neural network training, without considering the specific convolution kernel value, constitutes an effective strategy for utilizing the proposed OCPU. The performance enhancement achieved through the OCPU-

based convolutional layer is demonstrated in Supplementary Note 12.

Supplementary Note 2: Significant convolution results extraction

The process of 2D image flattening and significant convolution result extraction can be expressed with formulas. For the input image with $J \times K$ pixels, since the kernel size of the OCPU is 2×2, the input image can be divided into $(J-(2-1))=(J-1)$ sub-images by row, where each sub-image contains two rows of the input image (e.g., $2 \times K$ pixels for each sub-image). Then, each sub-image is flattened into a $1 \times 2K$ vector by column, and $(J-1)$ sub-images concatenated head-to-hail to form the $1 \times (2K \times (J-1))$ vector. After calculating with the OCPU, $1 \times (2K \times (J-1))$ convolution results are obtained in each channel, and the coordinates of significant values can be expressed as follows:

$$L = 2i + 2K(j-1), (i = 2,3,\ldots,K; j = 1,2,\ldots,J-1). \quad (11)$$

As shown in Supplementary Fig. 1, a 4×4 input image convolved with a 2×2 kernel in one channel is taken as an example. The input image is first divided into 3 sub-images where two adjacent rows are contained in each sub-image, and then these sub-images are flattened into three 1×8 vectors by flattening sub-images by column. Subsequently, three 1×8 vectors are concatenated head-to-hail and form a 1×24 vector to perform the convolution operation in the OCPU chip. According to Eq. (11), $J = K = 4$ and the significant values are $[y_4 \ \ y_6 \ \ y_8 \ \ y_{12} \ \ y_{14} \ \ y_{16} \ \ y_{20} \ \ y_{22} \ \ y_{24}]$ (marked with the orange background in Supplementary Fig. 1). The feature map can be obtained by reshaping these significant values into a 3×3 matrix $\begin{bmatrix} y_4 & y_6 & y_8 \\ y_{12} & y_{14} & y_{16} \\ y_{20} & y_{22} & y_{24} \end{bmatrix}$.

Supplementary Note 3: Convolution kernel experimental implementation and temporal waveforms

As shown in Fig. 5 in the main text, the first two non-negative kernels are realized with the first port of the OCPU, and the remaining kernels with real-value elements are realized by setting the fourth port as the reference port and then subtracting the reference port from the first output port. In the experiment, the actual transfer matrix of MMI M and phase shifters Φ cannot be independently measured since there are no reserved test ports in the OCPU design process. In the experiment, the required kernels are obtained by inputting an electrical pulse to the computing links and observing the output response. A schematic diagram of the input and output responses to realize five kernels is shown in Supplementary Fig. 2. Since a number of wavelength components are demultiplexed to the corresponding channels in the arrayed waveguide grating and each goes through the stated time delays and combined in the OCPU, the kernel of $\begin{bmatrix} 1 & 1 \\ 1 & 1 \end{bmatrix}$ is realized when the output pulse width is stretched four times (Supplementary Fig. 2(a)). Similarly, the kernel of $\begin{bmatrix} 1 & 0 \\ 0 & 1 \end{bmatrix}$ is achieved when two pulses without stretching are observed with the time interval of two data input periods (Supplementary Fig. 2(b)). For the three kernels with negative elements, such as $\begin{bmatrix} 1 & 0 \\ 0 & -1 \end{bmatrix}$, when the first output port outputs convolution kernel 2 and the fourth output port outputs an unstretched pulse whose amplitude is twice that of the first port, the kernel is realized by subtracting the fourth port from the first port (Supplementary Fig. 2(d)). Furthermore, the measurement of the splitting ratio at each output port is an alternative method to obtain the required kernels. By measuring the

splitting ratio, the elements of $|r_{uv}|^2$ in Eq. (5) are obtained, and the kernels can be calculated by subtracting the reference port from the other port as described in Eq. (10).

Simulations are simultaneously conducted to demonstrate the realization of five kernels. The matrix $M = \begin{bmatrix} -0.49-0.04i & 0.32+0.36i & -0.30-0.38i & -0.47-0.04i \\ 0.32+0.36i & -0.48-0.07i & -0.44-0.06i & -0.30-0.38i \\ -0.30-0.38i & -0.45-0.06i & -0.48-0.07i & 0.32+0.36i \\ -0.47-0.04i & -0.30-0.38i & 0.32+0.36i & -0.49-0.04i \end{bmatrix}$ is obtained from the simulation in Lumerical 2021 R2.5, and the required phase matrix Φ is gradually converged using the gradient descent algorithm. The eventual phase matrices for five kernels are shown in Supplementary Fig. 3, where the horizontal axis represents the number of phase shifters and the vertical axis represents the additional phase shift.

Supplementary Fig. 4 shows temporal waveforms obtained when performing optical convolution with the proposed OCPU. Supplementary Fig. 4(a-b) shows temporal waveforms when five digital images from the MNIST database are convolved with two kernels without negative elements. These temporal waveforms are obtained from one port of the proposed OCPU with a single acquisition. The feature images recovered from significant values of the temporal waveforms are shown below the temporal waveforms. Supplementary Fig. 4(c-e) shows temporal waveforms when five digital images are convolved with three kernels with negative elements. These temporal waveforms are obtained by subtracting the reference port from the other ports. Subtraction results in relatively large noise, which can be reduced by taking multiple acquisitions and averaging them. Temporal waveforms shown in Supplementary Fig. 4(c-e) and averaging 13 acquisitions to reduce noise and the corresponding feature images are shown below the temporal waveforms.

Supplementary Note 4: Convolution error and bit precision

To evaluate the performance of optical convolution with the proposed OCPU, the root mean square error (RMSE) between the convolutional result with the computer and the proposed OCPU is calculated. RMSE is the most common objective criterion used to evaluate the performance of images[2]. For the two-dimensional image, RMSE can be calculated as follows:

$$RMSE = \sqrt{\frac{1}{mn}\sum_{i=1}^{m}\sum_{j=1}^{n}\left(p_{ij} - q_{ij}\right)^2}, \tag{12}$$

where p_{ij} is the pixel value of the i-th row and the j-th column from the feature image with computer, q_{ij} is the pixel value of the i-th row and the j-th column from the feature image with the proposed OCPU, and m and n are the number of rows and columns of the feature image, respectively. For the image from the MNIST database with 28×28 pixels, the feature image obtained with a 2×2 kernel is 27×27 pixels; therefore, m and n in Eq. (12) is 27.

The bit precision N_b[3] is expressed as follows:

$$N_b = \log_2\left(\frac{\mu_{max} - \mu_{min}}{\sigma}\right). \tag{13}$$

where μ_{max} and μ_{min} are the maximum and minimum values of the output, respectively. σ is the standard deviation of the errors between the experimental output and expected output. The bit precision of the OCPU-based computing system mainly depends on the noise of the computing system and the stability of the OCPU. Assuming that the noise remains constant during the calculation, the impact on the calculation results mainly depends on the stability of the splitting ratio, which has no concern with the specific convolution kernel. Therefore, the feature images shown in Fig. 5 of the main text with two

kernels $\begin{bmatrix} 1 & 1 \\ 1 & 1 \end{bmatrix}$ and $\begin{bmatrix} 1 & -1 \\ -1 & 1 \end{bmatrix}$ are used to calculate the bit precision, corresponding to two cases of the OCPU to implement the convolution kernel: a single port for the non-negative convolution kernel and two ports for the real-valued convolution kernel. The results among 29160 MAC operations are shown in Supplementary Fig. 5, where the abscissa represents the expected output and the ordinate represents the measured output. The inset shows the histogram of the error distribution, revealing the standard deviation of $\sigma = -0.0298$. The calculation results are normalized to 1 so that $\mu_{max} = 1$ and $\mu_{min} = 0$, and the bit precision N_b is calculated as 5-bit.

Supplementary Note 5: Convolution kernel reconstruction ability

The reconfiguration of kernels based on the OCPU is not only in the phase shift but also in the intensities of incoherent light waves, where the cooperative work in both the amplitude and phase is able to substantially increase the reconfiguration of the convolution kernel. For serial convolution with the proposed OCPU, the 1D data are simultaneously modulated to the amplitudes of kernel wavelengths $\lambda_1 \sim \lambda_4$ with the same initial intensities. When taking the initial intensities of kernel wavelengths as an adjustable term, Eq. (1) can be rewritten as

$$I_w = \begin{bmatrix} w_1 i_1 \\ w_2 i_2 \\ w_3 i_3 \\ w_4 i_4 \end{bmatrix}. \tag{14}$$

The coefficient $w_u \left(u = 1 \sim 4 \right)$, which represents the intensity of every wavelength, can be combined with the transfer matrix R'. Then, the transfer matrix R' is modified as:

$$R_w = \begin{bmatrix} w_1 r_{11} & w_2 r_{12} & w_3 r_{13} & w_4 r_{14} \\ w_1 r_{21} & w_2 r_{22} & w_3 r_{23} & w_4 r_{24} \\ w_1 r_{31} & w_2 r_{32} & w_3 r_{33} & w_4 r_{34} \\ w_1 r_{41} & w_2 r_{42} & w_3 r_{43} & w_4 r_{44} \end{bmatrix}. \quad (15)$$

As seen from Eq. (15), the intensity is introduced into the kernel matrix R_w, and a new freedom dimension is added to increase the reconfigurable flexibility.

Supplementary Note 6: Computing speed improvement

Furthermore, the OCPU has high potential for scalable computing speed. The increasing number of input/output ports of the OCPU increases both the size and the number of kernels. In addition, wavelength-division multiplexing (WDM) can be introduced to further improve the computing speed. In Supplementary Fig. 6, we discuss how to further improve the computing speed.

When the data rate is set as f baud per second, the minimum wavelength interval is $2f$ to prevent sideband overlap. If the optical bandwidth is equal to BW, the total number of wavelengths available is $[BW \div 2f]$, where '[ ]' means round down. For an OCPU with N ports, $[[BW \div 2f] \div N]$ wavelengths are simultaneously fed into one input port, and the computing speed is inferred as:

$$S = [[BW \div 2f] \div N] \times N^2 \times f. \quad (16)$$

As we can see from Eq. (16), when BW, $2f$ and N meet an integer multiple relationship, the maximum computing speed is up to

$$S = \frac{BW \times N}{2}. \quad (17)$$

It is suggested that a 9×9 OCPU with a 3×3 kernel and the entire C-band (bandwidth of 4.375 THz) is involved, and the computing speed can reach $4.375 \times 9 \div 2 = 19.6875$ tera-MAC operations per second.

Another issue worth noting is the bandwidth of the PD. To sum the power of incoherent wavelengths using PD, the bandwidth of PD f_p, the data rate f and the minimum wavelength interval f_i of incoherent wavelengths fed to one PD need to meet the condition of $f_i - 2f > f_p$ to avoid the beat of sidebands between different wavelength components.

Supplementary Note 7: Simulation for 9×9 OCPU

Supplementary Fig. 7(a)-(b) shows the simulated optical field distribution of the 9×9 MMI with Lumerical from port 5 and port 1. The splitting ratios for nine input ports are shown in Supplementary Fig. 7(c), where the x-axis and the y-axis represent the numbers of input ports and output ports, respectively, and the z-axis represents the percentage of optical output.

Learning from the 4×4 architecture, the 9×9 OCPU chip comprises two 9×9 MMIs and 9 phase shifters. The size of the MMI from the input to output tapers is 144×25 μm^2, and the minimum footprint of the phase shifter considering the minimum space between phase shifters is 260×4 μm^2. As a result, the size of the 9×9 OCPU was 0.0166 mm^2.

The critical dimension of the silicon waveguide width for the multi-project wafer (MPW) foundry under ArF lithography is 25 nm. Therefore, the width of a 9×9 MMI is set as 25±0.025 μm in the simulation. Consequently, the mean errors in the splitting ratio range from 0.32% to 0.14%. Furthermore, the fabrication error of waveguide thickness is generally less than 10 nm, and the thickness of a 9×9 MMI is set as 220±10 nm. The simulated mean errors on the splitting ratio are 1.95% and 0.89%, respectively.

Taking the manufacturing error of the designed 9×9 OCPU into account, the

recognition accuracy is simulated on the handwritten digits (more details can be seen in Supplementary Note 12). From the simulation results, the influence of manufacturing error on recognition accuracy is small (less than 0.48%) and negligible.

Supplementary Note 8: Energy efficiency and compute density

The energy consumption for lasers to overcome shot noise and the capacitance of the photodetectors with fixed N_b bits precision is given by[4]

$$P_{lasers} \geq \frac{hv}{\eta} \max(2^{2N_b+1}, \frac{CV_d}{e}) f, \tag{18}$$

where hv is the photon energy for a center wavelength of 1550 nm, $C = 2.4$ fF and $V_d = 1$ V are the capacitance and driving voltage of the photodetectors[5], η is the combined quantum efficiency of the laser, photodetector, and optical link loss ($\eta \approx 40$ dB, assuming a 10% quantum efficiency of the laser and photodetector), e is the elementary charge, f is the data rate (16.60 Gbaud/s in the experiment). The total energy consumption of the computing system is written as

$$P_{total} = P_{lasers} + P_{DAC} + E_{mod} N_b f + NP_{ps} + NP_{ADC} \tag{19}$$

where N is the number of channels (set as 4 in 4×4 OCPU), and P_{DAC}, E_{mod}, N_{ps} and P_{ADC} are the energy consumption of the digital to analog converter (DAC), modulator, phase shifter and analog to digital converter (ADC), respectively. The figure of merit (FoM) for DAC is $FoM = 2^B \cdot f_s / P_{DAC}$, where B is the bit precision of DAC, f_s is the sample rate, and P_{DAC} is the energy consumption of DAC[6]. According to the reported DAC with a 14-bit precision, 10 GS/s sampling rate and 177 mW energy consumption[7], the DAC with the same FoM, 8-bit precision, and 25 GS/s sampling rate has an energy consumption of 6.91 mW. Here, the maximum energy consumption per phase shifter is 50 mW, and to

keep the required weight using four phase shifters, the average energy consumption P_{ps} is estimated to be 25 mW. The energy of the ADC and modulators are estimated as $P_{ADC} = 194$ mW/channel (Alphacore, A8B40G, 8-bit, 25 GHz) and $E_{mod} \approx 1$ pJ/b[8]. Therefore, in the case of N_b=5 and f=16.60 Gbaud/s, the total power is calculated as 1.28 W following Eq. (19). The maximum computing speed in four correlated non-negative kernels is calculated as 265.60 giga-MAC operations per second. Consequently, the efficiency is 4.84 pJ/MAC.

When considering the useless output from the proposed 4×4 OCPU, it will result in the reduction of energy efficiency. The relationship between the energy efficiency (E_n for non-negative kernels and E_r for real-value kernels) and the number N_o of useful output ports is expressed as

$$\begin{cases} E_n = \dfrac{P_{lasers} + P_{DAC} + E_{mod} N_b f + N P_{ps} + N_o P_{ADC}}{265.60G / 4 \times N_o} \\ E_r = \dfrac{P_{lasers} + P_{DAC} + E_{mod} N_b f + N P_{ps} + N_o P_{ADC}}{265.60G / 4 \times (N_o - 1)} \end{cases}. \tag{20}$$

The compute density, as another crucial evaluation criterion of chip performance, is defined as[4]

$$D = \frac{S(MACs / s)}{Area(mm^2)}. \tag{21}$$

The area of the MMI cell in the OCPU chip includes tapers with a footprint of 455 μm×15 μm, the area of each phase shifter considering the minimum space is 400 μm×4.5 μm, and the total area of the OCPU chip is 0.0209 mm^2. The computed density is then computed as 12.74 TMACs/s/mm^2.

The energy efficiency and computational density can be further improved by scaling the OCPU chip as well as drawing into the WDM technology. As discussed in Note 6,

scaling into 9 channels at a data rate of 24.31 Gbaud/s, 10 wavelengths are multiplexed in each channel, and the maximum computing speed is 19.6875 tera-MAC operations per second with 90 multiplexed paths. The chip size is expected to be 0.0166 mm^2 in the high-index silicon platform. For the energy consumption, the energy consumption is calculated as 18.77 W when the average energy consumption of the SOI-based thermo-optic phase shifter is 1 mW (2 mW/π for each SOI-based thermo-optic phase shifter). Ultimately, the efficiency is 0.95 pJ/MAC, and the computational density is 1.19 PMACs/s/mm^2.

Supplementary Note 9: Relationship between bit precision and recognition accuracy

The relationship between bit precision and recognition accuracy on the MNIST database is simulated in the MNIST dataset, where 60000 images in the training set are utilized for training and 10000 images in the test set are used for testing. In the simulation, a CNN using the structure shown in Fig. 6(a) in the main text is constructed with a convolutional layer and a fully connected layer, in which two 2×2 kernels are kept at ($\begin{bmatrix} 0.6003 & -0.4199 \\ -0.3902 & 0.2098 \end{bmatrix}$, $\begin{bmatrix} 0.0458 & -0.3413 \\ 0.2988 & -0.0032 \end{bmatrix}$) and remain unchanged from the experimental kernels. According to Eq. (13), a variety of Gauss noises are attached to the convolution result to remark on the corresponding bit precisions. The variation in recognition accuracy with bit precision is shown in Supplementary Fig. 8, where the gray background is the fluctuation range of the final recognition accuracy rate in ten training sessions and the blue line indicates the average recognition accuracy rate. The simulation results show that the recognition accuracy increases with increasing bit precision.

Supplementary Note 10: Analysis of recognition accuracy

In the experiment, to guarantee that the SOA works in the linear amplification region, the optical power input to the SOA and output from the SOA is relatively low, and the optical power input to the PDs is only approximately -1 dBm, which results in a low signal-to-noise ratio after photoelectric conversion. The process of analog-to-digital conversion in the OSC and the process of subtraction in the digital domain further degraded noise performance. The relatively high noise results in 5-bit precision, and the limited number and size of kernels result in relatively low accuracy.

Two measures can be taken to improve the recognition accuracy. On the one hand, a balanced detection method can be adopted to reduce the noise and improve the bit precision. By introducing an optical coupler in the reference port to divide the reference signal into several beams, balanced photodetectors (BPDs) can be utilized between the reference signals and other signals. With BPD, the process of subtraction is accomplished in the analog electrical domain, and the same link noise can be eliminated. Fewer analog-to-digital conversions also avoid the superposition of noise introduced by analog-to-digital conversions in the subtraction process. In addition, SOAs with a larger linear amplification region and higher output power can also improve the output power. On the other hand, the limited number and size of kernels (2 2×2 kernels here) results in relatively low recognition accuracy, and by optimizing the structure of the neural network, such as increasing the number and expanding the size of convolution kernels, the recognition accuracy can be further improved[9].

Supplementary Note 11: The limitations of convolution kernels

From Eq. (9) and (10), the formed $n-1$ kernels are exactly correlated to others and

cannot be independently adjusted for a $n \times n$ OCPU. All these phase shifters are employed to implement the reconfiguration of one kernel, and simultaneously, the remaining kernels are changed accordingly. Despite the limited reconfigurability, the proposed OCPU architecture can work as a high-performance convolutional layer. The simulation results are shown in Supplementary Note 12 to illustrate the performance of the 9×9 OCPU with the benchmark of the MNIST database.

In addition, for one of the convolution kernels realized with the OCPU, the possible kernels can take are simulated. Two output ports are utilized to realize these kernels, and 2×2 kernels realized with 4×4 OCPU and 3×3 kernels with 9×9 OCPU are simulated. For the proposed OCPU architecture, the transfer matrix M for the MMI cell is fixed once the chip is fabricated, and the tunable elements are the phase shifters represented by Φ. In the process of simulation, the transfer matrix M for the MMI cell is obtained from the simulation using Lumerical. The target kernel is first generated, and then the additional phase introduced by the phase shifter is optimized using the gradient descent algorithm, and the output of each port of the OCPU is normalized to match the target convolution kernel.

For an $n \times n$ convolution kernel, if each element can take p possible values, there are $p^{(n \times n)}$ kernels. Therefore, a great number of possible values for elements in real-valued kernels will result in a significant increase in the computational demand (e.g., for weight precision of 5-bit, $p = 2^5 = 32$, there will be 32^4 kernels for $n = 2$ and 32^9 kernels for $n = 3$). To reduce hardware requirements and consider computing ability, kernels involving only -1,0,1 are simulated to illustrate the kernels achievable.

For the 4×4 OCPU, there are a total of 3^4=81 kernels, and for the 9×9 OCPU, there are a total of 3^9=19683 kernels. After optimization, the maximum bias, which is defined as the absolute value of the maximum error between elements in the target kernels and generated kernels, is recorded and shown in Supplementary Fig. 9. The target kernel is successfully represented when the maximum bias is lower than 0.0313 (corresponding to a weight adjustment precision of 5-bit). 100% 2×2 kernels are represented with the 4×4 OCPU (Supplementary Fig. 9(a)), and 99.09% 3×3 kernels are successfully represented with the 9×9 OCPU (Supplementary Fig. 9(b)).

The simulation results show that the arbitrary reconfiguration of one 2×2 kernel involving -1,0,1 with the 4×4 OCPU can be realized. Scale expansion does have an impact on OCPU reconfigurability, but the impact is weak (the representable kernels are reduced by 0.91% when expanding from 4×4 to 9×9).

Supplementary Note 12: Handwritten digit recognition with 9×9 OCPU

The 9×9 OCPU is simulated to conduct a convolutional layer with 8 3×3 correlated real-valued kernels. Kernels in the convolutional layer are generated according to the extended Eq. (10) (3 2×2 kernels extend to 8 3×3 kernels). The 9×9 transfer matrix is exported from the simulation in Lumerical, and the fifth output port is selected as the reference port. After the convolution operation, according to Eq. (13), the 5-bit precision was characterized by adding Gaussian noise to the convolution results, and the simulation accuracy reached 96.35% on the MNIST database. The recognition accuracy in 5000 epochs of training is shown in Supplementary Fig. 10.

The influence of manufacturing errors on recognition accuracies is also verified by

simulation, and the simulation accuracies are 96.45% @ (25.025 μm wide, 220 nm thick), 96.39% @ (24.975 μm wide, 220 nm thick), 95.87% @ (25 μm wide, 230 nm thick), and 96.02% @ (25 μm wide, 230 nm thick). The simulation results proved the negligible influence of manufacturing errors (accuracy error less than 0.48%) and the high robustness of the proposed 9×9 OCPU.

To evaluate the performance of the OCPU-based convolutional layer, two neural networks, a single layer fully connected network and a general CNN, are simulated. The fully connected network only contains a fully connected layer, and the CNN consists of a convolutional layer and a fully connected layer, where 8 general 3×3 kernels are contained in the convolutional layer. Similarly, Gaussian noise of 5-bit is added to the convolution results of the general CNN. The recognition accuracies of 92.87% for the fully connected network and 98.31% for the CNN are shown in Supplementary Fig. 10. Although the OCPU-based convolutional layer shows lower recognition accuracy than the general convolutional layer (1.96%), it can still work as a convolutional layer with high performance and low power consumption.

Supplementary Note 13: Electrical overhead for the CNN

The electrical postprocessing in the proposed OCPU-based CNN is divided into three parts: the extraction of significant values, nonlinear activation and full connection. The processes of subtraction and averaging are also contained for real-valued kernels. The number of operations in each process for non-negative and real-valued kernels are shown in Supplementary Table 1. The process of significant data extraction takes 1458 operations for two 2×2 non-negative kernels and 18954 operations for two 2×2 real-valued kernels. In

addition, for two 2×2 real-valued kernels, the additional subtraction and average processes contain 39312 and 18954 operations, respectively. For both non-negative and real-valued kernels, the process of nonlinear activation with the ReLU function requires 1458 operations, and the process of full connection requires 29160 operations. For a number of the reported optical CNN architectures[10-12], these two processes are generally accomplished with electrical hardware. The OCPU adds three processes to this, namely, significant value extraction, subtraction and averaging. Therefore, with the sum operations of nonlinear activation and full connection as the baseline, the additional processes append 4.76% electrical overhead for non-negative kernels (significant value extraction) and 252.20% electrical overhead for real-valued kernels (significant value extraction, subtraction and average).

Since optical computing shows some advantages in terms of efficiency and computational density, more electrical overhead inevitably affects the overall performance of the OCPU-based CNN system. Therefore, it is crucial to minimize the additional electrical overhead to fully leverage the benefits of optical computing. The process of significant value extraction is caused by the serial data input method, which is also needed in other serial data input works[13] and can be avoided by inputting data in a parallel method with several modulators. The operation of subtraction and averaging can be avoided by optimizing the computing link, which has been discussed in Note 10.

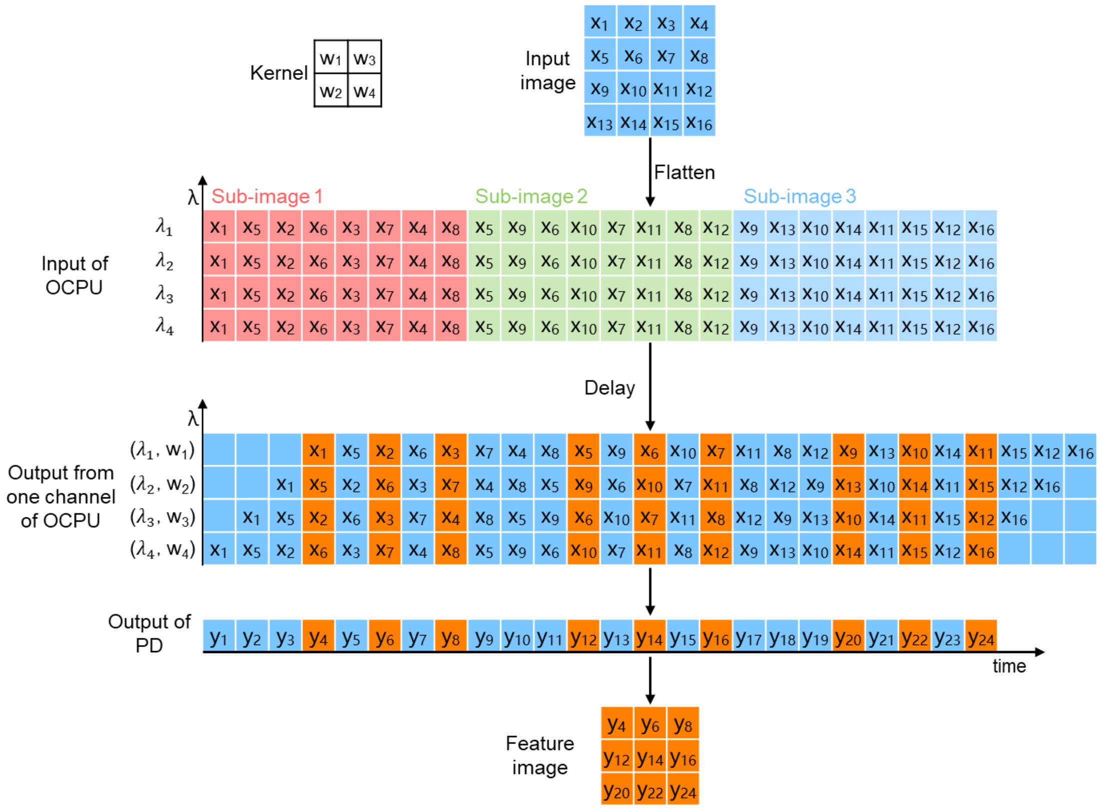

Supplementary Fig. 1 The process of significant convolution result extraction.

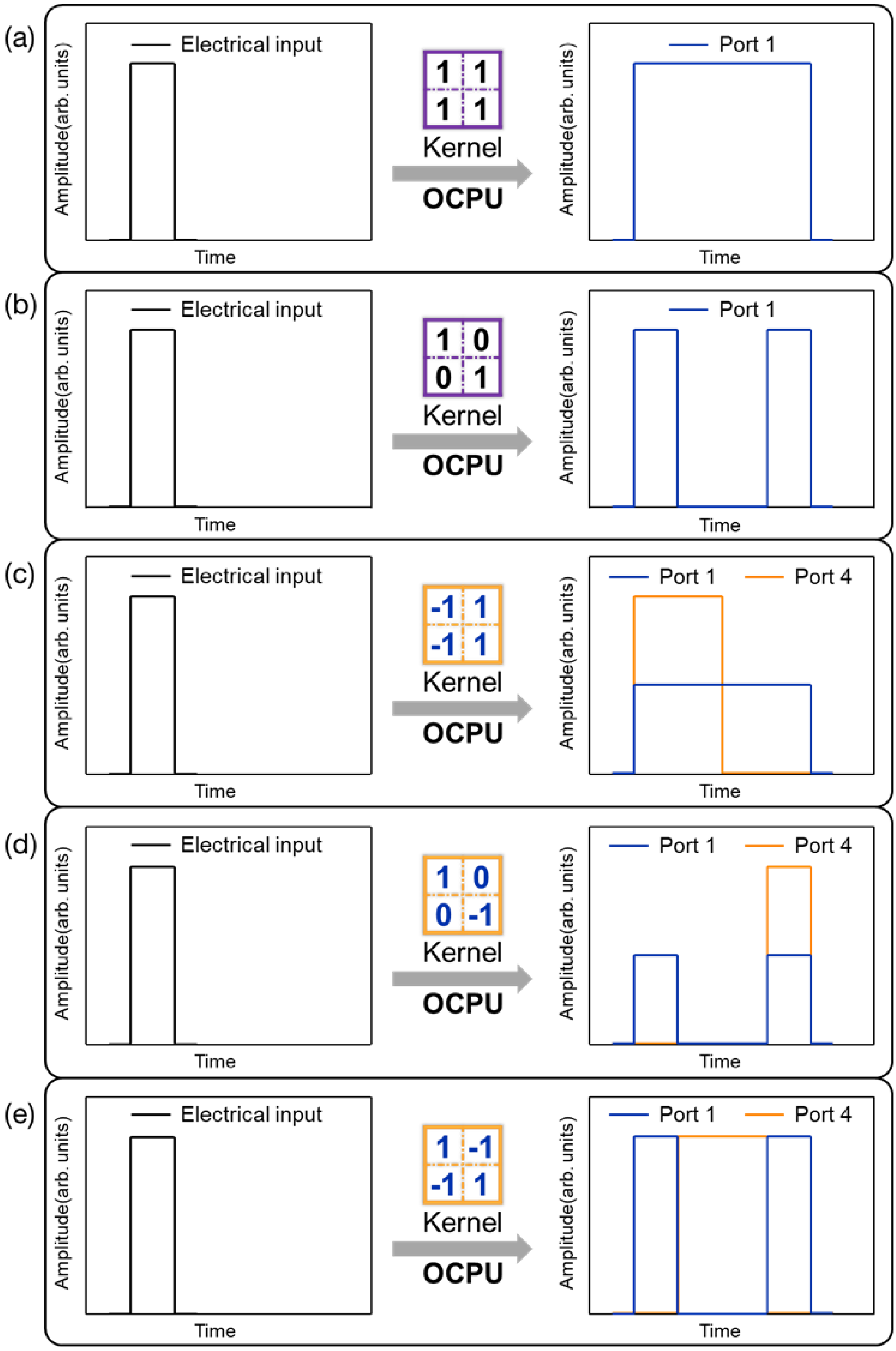

Supplementary Fig. 2 Schematic diagram of the input and output responses to realize five kernels (corresponding to (a-e) respectively).

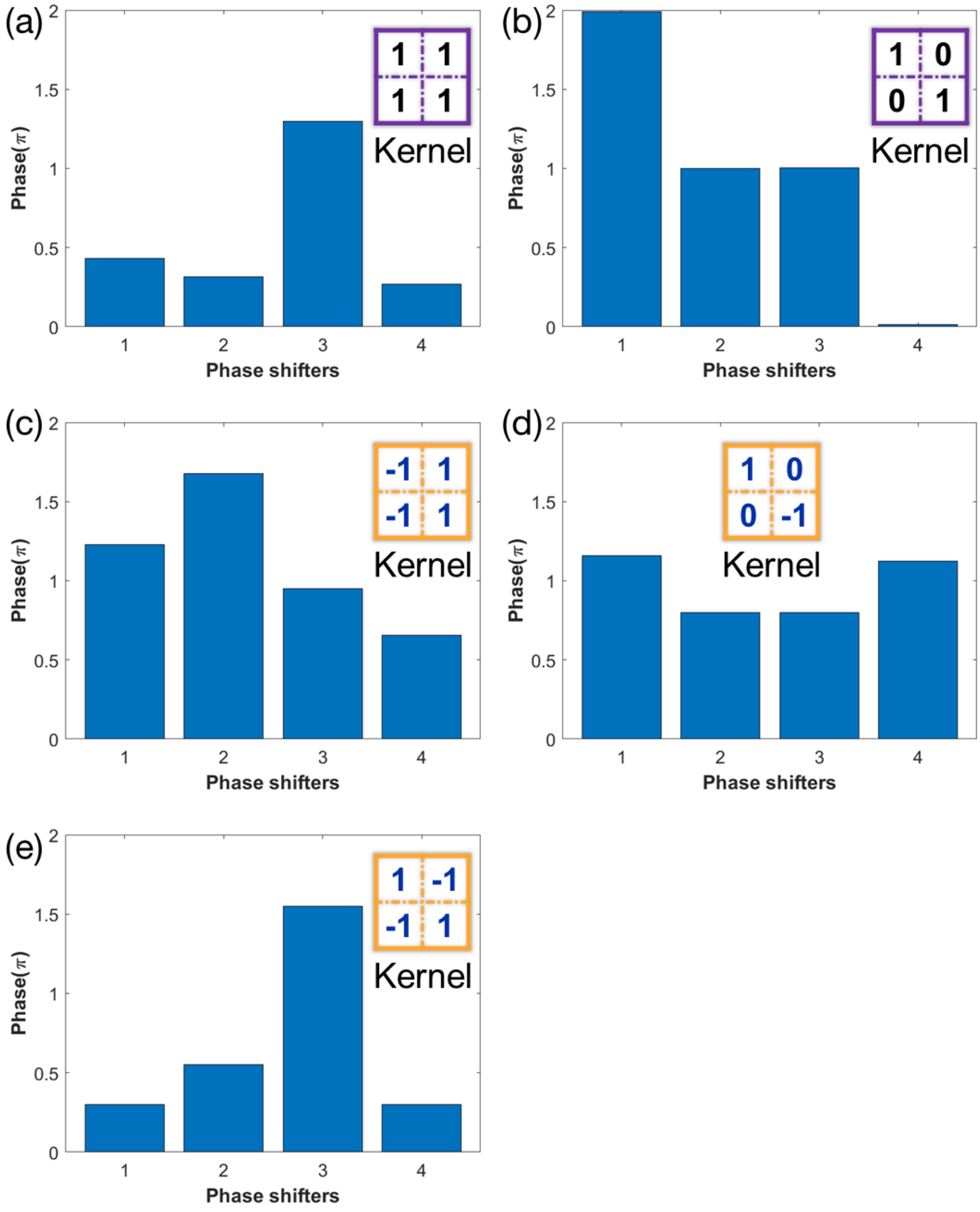

Supplementary Fig. 3 The required phase to realize five kernels (corresponding to (a-e) respectively).

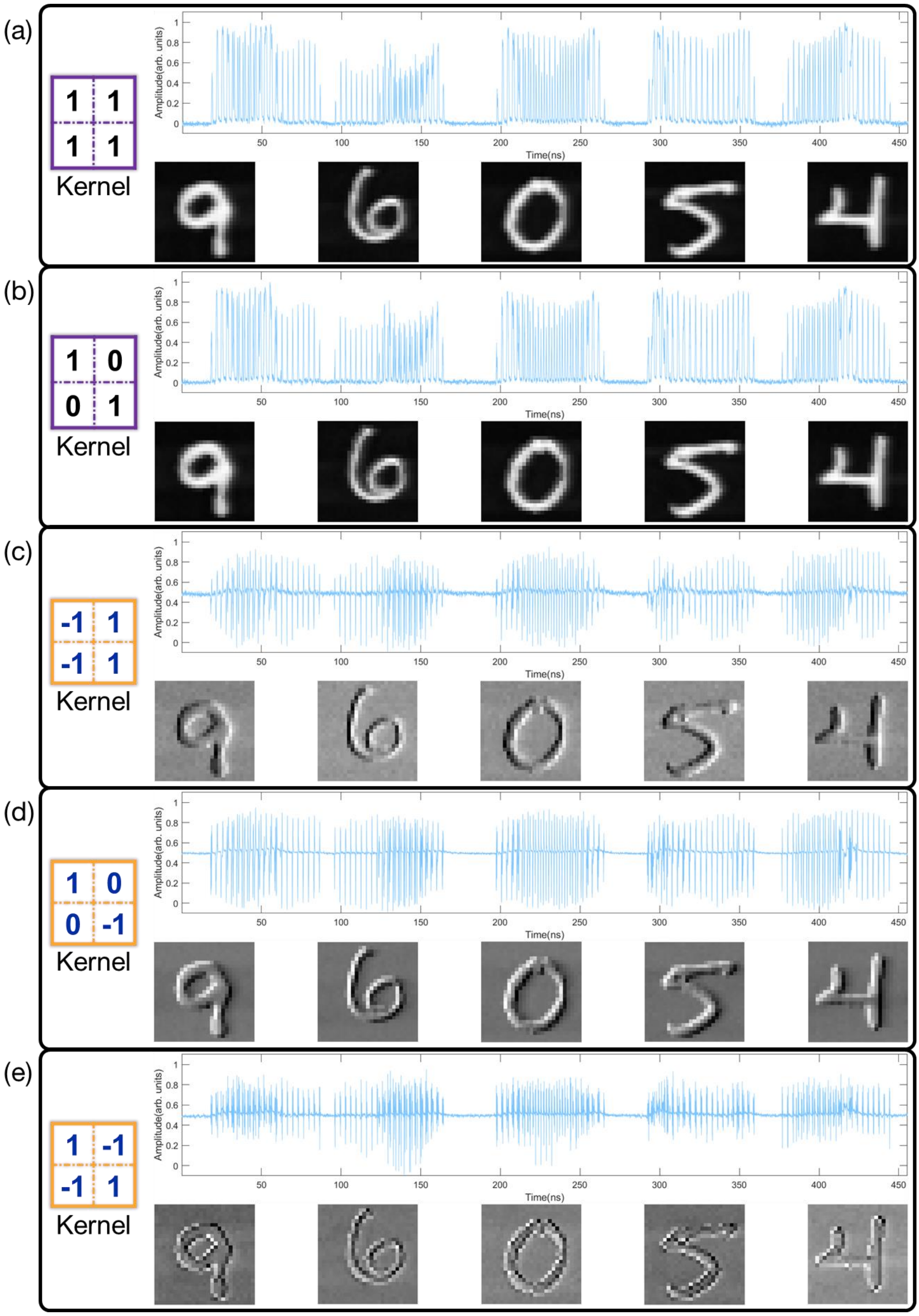

Supplementary Fig. 4 The temporal waveform obtained with the OCPU when performing the convolution operation with five kernels (corresponding to (a-e) respectively).

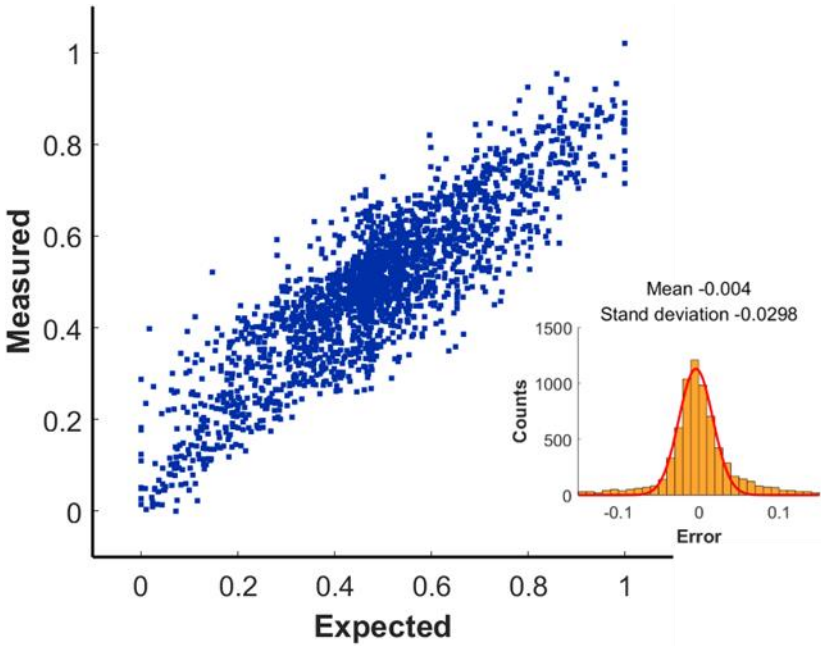

Supplementary Fig. 5 Calculation accuracy for 29160 MAC operations. The inset shows the histogram of the data revealing a standard deviation of -0.0298 and therefore a precision of 5-bit.

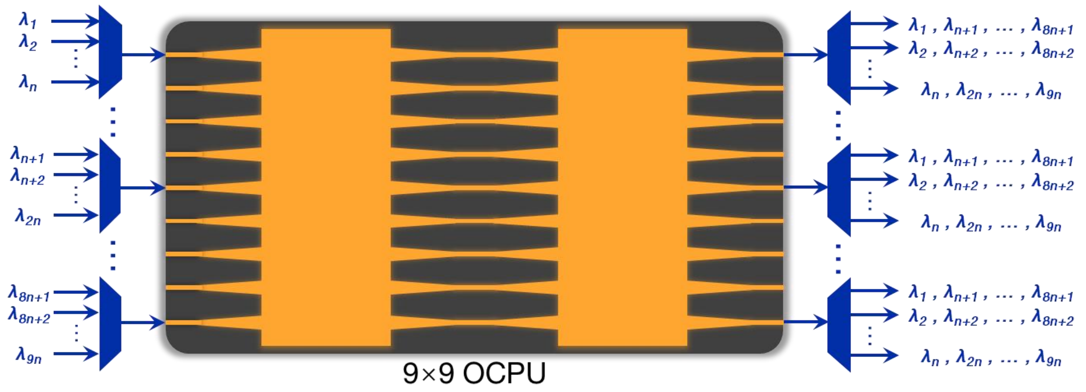

Supplementary Fig. 6 The realization of wavelength division multiplexing in each input port of the OCPU to improve the computing speed.

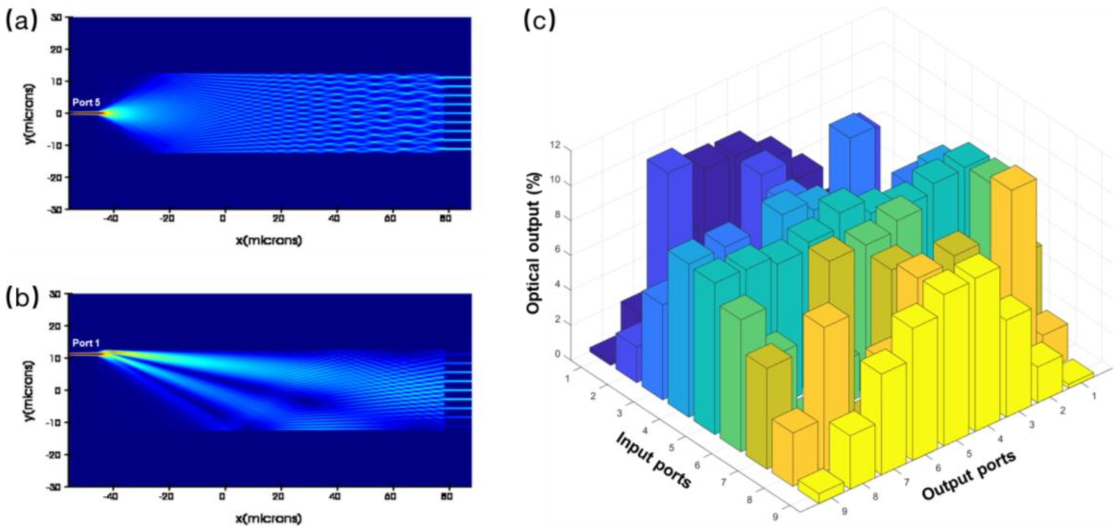

Supplementary Fig. 7 The simulated optical splitting ratio of the 9×9 MMI. (a)-(b) Optical field distribution of the 9×9 MMI when input from port 5 and port 1. (c) The splitting ratios for nine input ports.

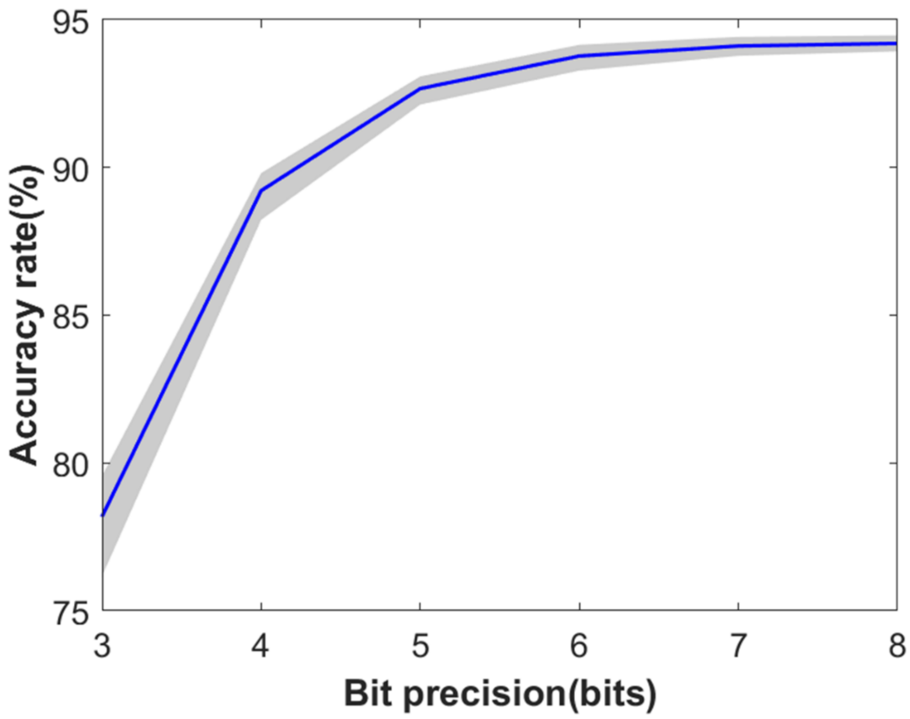

Supplementary Fig. 8 Variation in recognition accuracy with bit precision.

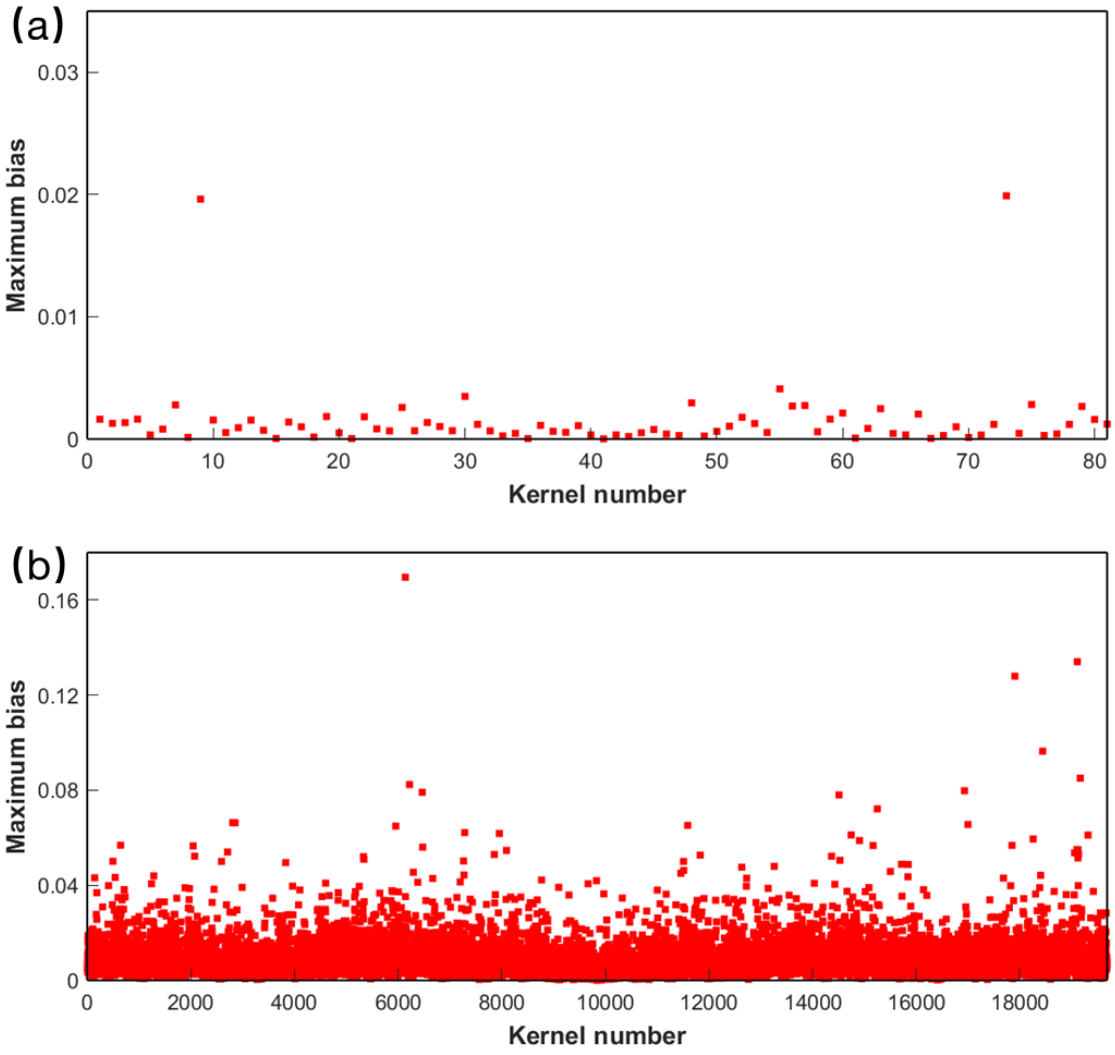

Supplementary Fig. 9 The maximum bias for the kernel realized with the 4×4 OCPU (a) and 9×9 OCPU (b).

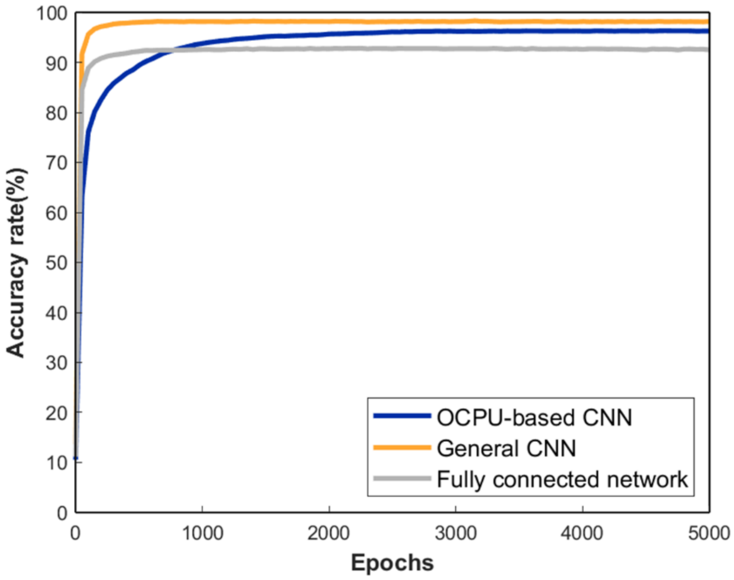

Supplementary Fig. 10 Handwritten digit recognition accuracy with OCPU-based CNN, general CNN and fully connected network.

Process	Output shape	Operations	Operations in Formulas	Scaling[a]
Significant value extraction	729×2	1458	$N_0(J-1)(K-1)$	5.00%
Nonlinear activation (ReLU)	1×1458	1458	$N_0(J-1)(K-1)$	5.00%
Fully connection	1458×10	29160	$20N_0(J-1)(K-1)$	100.00%
Total		32076	$22N_0(J-1)(K-1)$	110.00%

(a) Non-negative kernels.

Process	Output shape	Operations	Operations in Formulas	Scaling[a]
Subtraction	1512×13×2	39312	$26N_0K(J-1)$	134.81%
Significant value extraction	729×13×2	18954	$13N_0(J-1)(K-1)$	65.00%
Average	729×2	18954	$13N_0(J-1)(K-1)$	65.00%
Nonlinear activation (ReLU)	1×1458	1458	$N_0(J-1)(K-1)$	5.00%
Fully connection	1458×10	29160	$20N_0(J-1)(K-1)$	100.00%
Total		107838	$N_0(J-1)(73K-47)$	369.81%

(b) Real-valued kernels.

Supplementary Table 1. Electrical operations for non-negative kernels and real-valued kernels.

[a] The data in the scaling column are normalized to the number of operations in the fully connected layer.

Supplementary References

1 Horn, R. A. in *Proc. Symp. Appl. Math.* 87-169 (1990).

2 Eskicioglu, A. M. & Fisher, P. S. Image quality measures and their performance. *IEEE Trans. Commun.* **43**, 2959-2965 (1995).

3 Tait, A. N. *et al.* Feedback control for microring weight banks. *Opt. Express* **26**, 26422-26443 (2018).

4 Nahmias, M. A. *et al.* Photonic multiply-accumulate operations for neural networks. *IEEE J. Sel. Top. Quantum Electron.* **26**, 7701518 (2020).

5 Chen, L. & Lipson, M. Ultra-low capacitance and high speed germanium photodetectors on silicon. *Opt. Express* **17**, 7901-7906 (2009).

6 *An electro-photonic system for accelerating deep neural networks* (2021).

7 Huang, H. Y., Chen, X. Y. & Kuo, T. H. A 10-gs/s nrz/mixing dac with switching-glitch compensation achieving sfdr >64/50 dbc over the first/second nyquist zone. *IEEE J. Solid-State Circuits* **56**, 3145-3156 (2021).

8 Miller, D. A. B. Device requirements for optical interconnects to silicon chips. *Proc. IEEE* **97**, 1166-1185 (2009).

9 Xu, X., Zhu, L., Zhuang, W., Lu, L. & Yuan, P. A convolution neural network implemented by three 3×3 photonic integrated reconfigurable linear processors. *Photonics* **9**, 80 (2022).

10 Bangari, V. *et al.* Digital electronics and analog photonics for convolutional neural networks (deap-cnns). *IEEE J. Sel. Top. Quantum Electron.* **26**, 7701213 (2020).

11 Feldmann, J. *et al.* Parallel convolutional processing using an integrated photonic tensor core. *Nature* **589**, 52-58 (2021).

12 Xu, S., Wang, J., Wang, R., Chen, J. & Zou, W. High-accuracy optical convolution unit architecture for convolutional neural networks by cascaded acousto-optical modulator arrays. *Opt. Express* **27**, 19778-19787 (2019).

13 Xu, X. *et al.* 11 tops photonic convolutional accelerator for optical neural networks. *Nature* **589**, 44-51 (2021).
